# Oxidative and salt stresses alter the 26S proteasome holoenzyme and associated protein profiles in *Arabidopsis thaliana*

**DOI:** 10.1186/s12870-021-03234-9

**Published:** 2021-10-25

**Authors:** Diana Bonea, Jenan Noureddine, Sonia Gazzarrini, Rongmin Zhao

**Affiliations:** 1 Department of Biological Sciences, University of Toronto, 1265 Military Trail, Toronto, ON M1C 1A4 Canada; 2 Department of Cell and Systems Biology, University of Toronto, 25 Harbord Street, Toronto, ON M5S 3G5 Canada

**Keywords:** 26S proteasome, Abiotic stresses, Assembly chaperone, Molecular chaperone, Plant stress response, Proteasome assembly, Protein homeostasis, Protein degradation, PBAC1

## Abstract

**Background:**

The 26S proteasome, canonically composed of multi-subunit 19S regulatory (RP) and 20S core (CP) particles, is crucial for cellular proteostasis. Proteasomes are re-modeled, activated, or re-localized and this regulation is critical for plants in response to environmental stresses. The proteasome holoenzyme assembly and dissociation are therefore highly dynamic in vivo. However, the stoichiometric changes of the plant proteasomes and how proteasome associated chaperones vary under common abiotic stresses have not been systematically studied.

**Results:**

Here, we studied the impact of abiotic stresses on proteasome structure, activity, and interacting partners in *Arabidopsis thaliana*. We analyzed available RNA expression data and observed that expressions of proteasome coding genes varied substantially under stresses; however, the protein levels of a few key subunits did not change significantly within 24 h. Instead, a switch in the predominant proteasome complex, from 26S to 20S, occurs under oxidative or salt stress. Oxidative stress also reduced the cellular ATP content and the association of HSP70-family proteins to the 20S proteasome, but enhanced the activity of cellular free form CP. Salt stress, on the other hand, did not affect cellular ATP level, but caused subtle changes in proteasome subunit composition and impacted bindings of assembly chaperones. Analyses of an array of T-DNA insertional mutant lines highlighted important roles for several putative assembly chaperones in seedling establishment and stress sensitivity. We also observed that knockout of PBAC1, one of the α-ring assembly chaperones, resulted in reduced germination and tearing of the seed coat following sterilization.

**Conclusions:**

Our study revealed an evolutionarily conserved mechanism of proteasome regulation during oxidative stress, involving dynamic regulation of the holoenzyme formation and associated regulatory proteins, and we also identified a novel role of the PBAC1 proteasome assembly chaperone in seed coat development.

**Supplementary Information:**

The online version contains supplementary material available at 10.1186/s12870-021-03234-9.

## Background

Cellular protein homeostasis depends in large part on protein degradation that occurs within both cytosol and major membrane-bound organelles. In eukaryotes, the majority of non-lysosome/vacuole proteolysis takes place through the highly conserved ubiquitin proteasome system (UPS) in which the macro protein complex proteasome degrades proteins that are primarily labeled with polyubiquitin chains [25, 61, 69]. Central to the proteasome is the 20S core particle (CP), made up of two outer α-rings, and two inner β-rings each of which contains three catalytically active subunits [30]. While some cellular 20S proteasomes exist in free-floating and primarily latent forms [53, 55], most are capped at one or both ends by the 19S regulatory particles (RP), thus forming a 26S proteasome which is capable of selectively recognizing, unfolding and degrading polyubiquitinated substrate proteins in an ATP-dependent manner [4, 11, 22]. On the other hand, the 20S CP could also be capped by the regulator PA200/Blm10 during the CP cellular transport or under stress conditions [76].

Plants are sessile and must adapt to and develop in fluctuating environmental conditions. Such a feat is possible partly due to the complicated and stringently regulated UPS that is involved in most cellular processes including embryogenesis, growth, senescence, hormone signalling and abiotic stress response [60, 61]. Genomics data have shown that the plant *Arabidopsis thaliana* contains approximately 1600 UPS-related genes, most of which encode proteins for E3 enzymes that are responsible for substrate polyubiquitination [69, 70, 77]. It is therefore not surprising that the polyubiquitination pathway has been extensively studied [48, 61, 64]. However, recent studies in yeast and animals showed that the 26S proteasome macromolecule structure is highly dynamic, and its controlled and modular assembly may play vital roles in regulating protein degradation in rapid response to stresses [11, 24].

In both yeast and mammals, 20S proteasome levels can be immediately increased via dissociation of the 26S proteasome. This may be facilitated by elevated ROS production [43], by helper proteins Ecm29 or Hsp70 [31, 73], or via post-translational modifications [8, 65, 75]. In the long term, a higher cellular ratio of 20S/26S proteasome can be achieved through the de novo assembly of more 20S CP relative to 19S RP. This, in turn, could be accomplished by fine-tuning the abundance and/or activity of proteasome assembly chaperones [32, 59]. Indeed, overexpression of the β-ring assembly chaperone UMP1 in yeast or hUMP1/POMP in human cells leads to increased proteasome activity and enhanced cell viability following treatment with oxidants [12, 16].

Plants are perhaps even more susceptible to oxidants than yeast and mammals, since they generate ROS by-products not only from aerobic respiration in mitochondria [50], but also from peroxisomes, chloroplasts and plasma membrane-located NADPH oxidase [21]. Any abiotic stress that induces stomatal closure and limits CO_2_, such as drought, salinity and temperature extremes, can exacerbate ROS production beyond the scavenging capacity of the cell, leading to damage of various biomolecules, especially proteins [17, 20]. Like those in yeast and mammals, the 20S proteasome in plant also appears to play an important role under oxidative stress. In maize, sugar deprivation induces a mild oxidative stress, which increases 20S proteasome activity via carbonylation of its subunits [6]. 20S proteasome subunits in tobacco are both transcriptionally and translationally induced upon defense elicitation and the burst of ROS [66]. In Arabidopsis, elevated temperature during seed germination inhibits the UPS system and alters the stoichiometry of 26S proteasome, leading to thermoinhibition [14]. It is evident that 26S proteasomes are dynamically regulated at all stages of the plant life cycle and in response to various stresses [47, 77].

Altered expression of individual proteasome subunits or associated proteins have also been shown to impact the dynamics of cellular proteasomes. Loss-of-function mutations in *Arabidopsis* 19S RP subunits RPT2a, RPN10, and RPN12a lead to a decline in 26S while increasing 20S proteasomes and ubiquitin-independent degradation [38]. Due to changes in proteasome stoichiometry and activity, these mutants are more tolerant of oxidative stresses induced by methyl viologen or H_2_O_2_. Additionally, RP subunit mutants like *rpn1a*, *rpn10-1* are hypersensitive to high salinity stress [63, 72]. Loss of PBE1, one of the two β5 paralogues in 20S CP, negatively impacts CP assembly and results in seedlings that are hypersensitive to salt stress [33]. Additionally, *ump1a* and *ump1b* seedlings, the two CP β-ring assembly chaperone mutants [27], as well as the α-ring assembly chaperones *pbac1, pbac2,* and *pbac5* [45] display hypersensitivity to salt and osmotic stress. Although all these studies suggest that boosting 20S proteasome levels could be an adaptive response to oxidative or salt stress, analysis of stoichiometric changes of the proteasomes under abiotic stresses in plants have not been systematically studied.

Here, we examined the structural and functional adaptions of the plant proteasome in response to osmotic, salinity and oxidative stresses, as well as accessory factors that may help mediate these changes. As reported in yeast and mammalian cells, our results demonstrate a dissociation of the *Arabidopsis* 26S proteasome under salt and oxidative stresses. Under oxidative stress, a deficiency in cellular ATP level and decreased association of HSP70-family chaperones with the proteasome was observed. In contrast, salt stress affected proteasome binding to assembly chaperones or alternate caps, including PA200. Our genetic analysis also suggests a role for assembly chaperones *PBAC*, *UMP1,* and *HSM3* in regulating the transition from seed to seedling, as well as a new positive role for the plant α-ring assembly chaperone PBAC1 during oxidative stress in seeds.

## Results

### Salt and oxidative stresses alter the 26S proteasome stoichiometry while having little effect on the total amount of 20S proteasome

**Fig. 1:**
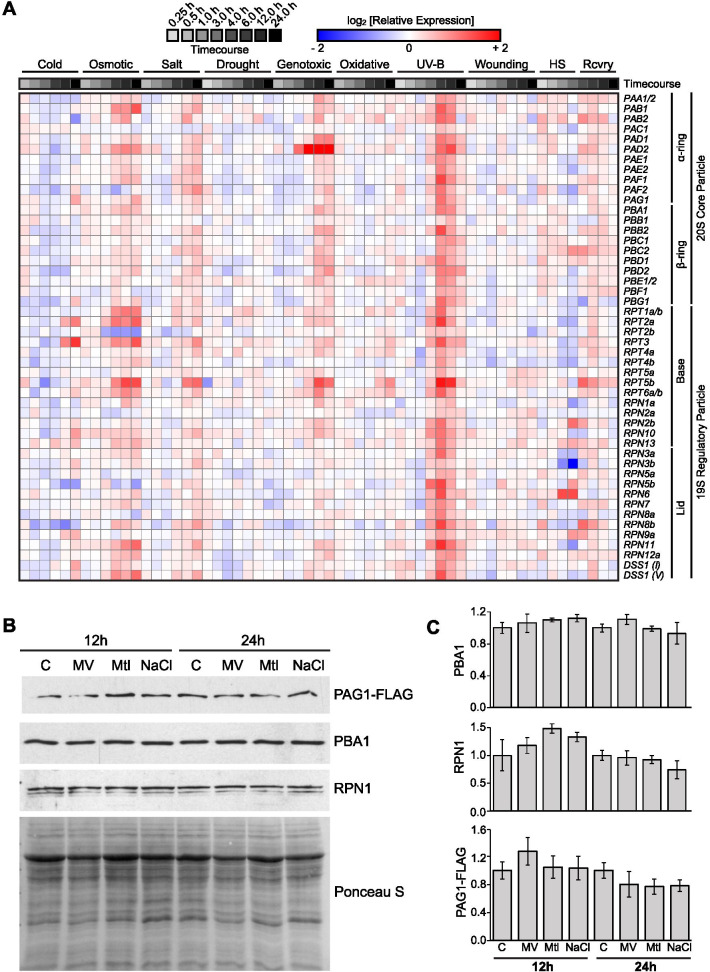
Effect of abiotic stresses on expression and steady-state level of proteasome subunits. **A** Microarray data of Arabidopsis seedlings exposed to cold (4 °C), osmotic (300 mM mannitol), salt (150 mM NaCl), drought (air stream), genotoxic (1.5 μg/ml bleomycin), oxidative (10 μM methyl viologen), UV-B, wounding, and heat shock (38 °C) stresses from Kilian et al. [37] were obtained via the BAR Expression Browser (http://bar.utoronto.ca/) and organized into a heat map using Morpheus (broadinstitute.org). Heat map displays log_2_-transformed expression-fold changes with min/max cut-off values of − 2/+ 2. Note that for heat stress, the 4.0- to 24.0-h time points represent variable 25 °C recovery times (Rcvry) following an initial 3.0-h heat shock (HS) at 38 °C. **B** Eight-day-old *PAG1-FLAG* seedlings were exposed to 10 μM methyl viologen (MV), 300 mM mannitol (Mtl), and 150 mM NaCl for 12 and 24 h. Anti-FLAG, anti-PBA1, and anti-RPN1 immunoblots of *PAG1-FLAG* total lysates were resolved on 12% SDS-PAGE, highlighting representative subunits of the CP α-ring, β-ring, and RP lid, respectively. Loading was normalized based on fresh weight and a Ponceau S stain is included as a loading control. It should be noted that the overall protein profiles as revealed by Ponceau S staining in **B** for either 12- or 24-h treatments are comparable suggesting viable seedlings. Three biological replicates were performed, and quantification of protein levels is shown in (**C**)**,** and no significant change was observed

A survey of publicly-available microarray data [37, 68] showed that abiotic stresses altered the expression profile of most 20S and 19S proteasome subunit genes in *Arabidopsis* (Fig. 1A). Many of the proteasome subunits are encoded by two paralogous genes and they can be very differently expressed under the same type of abiotic stress. For example, *RPT2a* is induced while *RPT2b* is repressed under osmotic stress, and *RPT5b* is strongly induced while *RPT5a* is only slightly upregulated under various abiotic stresses. This suggests that some paralogs might be preferentially incorporated over others under certain stresses to assemble different proteasome isotypes.

To test this hypothesis, we used a previously generated *pag1-1*/*pPAG1:PAG1-FLAG* line, which allows the isolation of proteasomal complexes in *Arabidopsis* [7]. Out of all stresses (Fig. 1A), osmotic and salt stresses were chosen due to their high relevance to natural stress conditions. Oxidative stress was also investigated since both osmotic and salt stresses induce a secondary oxidative stress response. Ten-day-old wild type and *pag1-1*/*pPAG1:PAG1-FLAG* seedlings grown on non-stress MS media were transferred to 300 mM mannitol, 150 mM NaCl or 10 μM methyl viologen (MV) for 12 and 24 h (Supplementary Fig. S1). We chose 12 and 24 h because transient exposure of seedlings to these concentrations of NaCl, mannitol and MV induced changes in transcript levels of most proteasomal subunit genes ([37]; Fig. 1A). The relative amounts of 26S proteasome under these stresses were first examined for the representative CP subunits PBA1 and PAG1, which are both encoded by a single gene in Arabidopsis, and the RP subunit RPN1. Our immunoblots indicated that none of these three proteins experienced significant change at the protein level (Fig. 1B and C), suggesting their altered expressions at the RNA levels (Fig. 1A) did not translate into significant change at the protein level within 24 h after stress treatment, and may not readily affect the total amounts of cellular 26S proteasome at this stage.

**Fig. 2:**
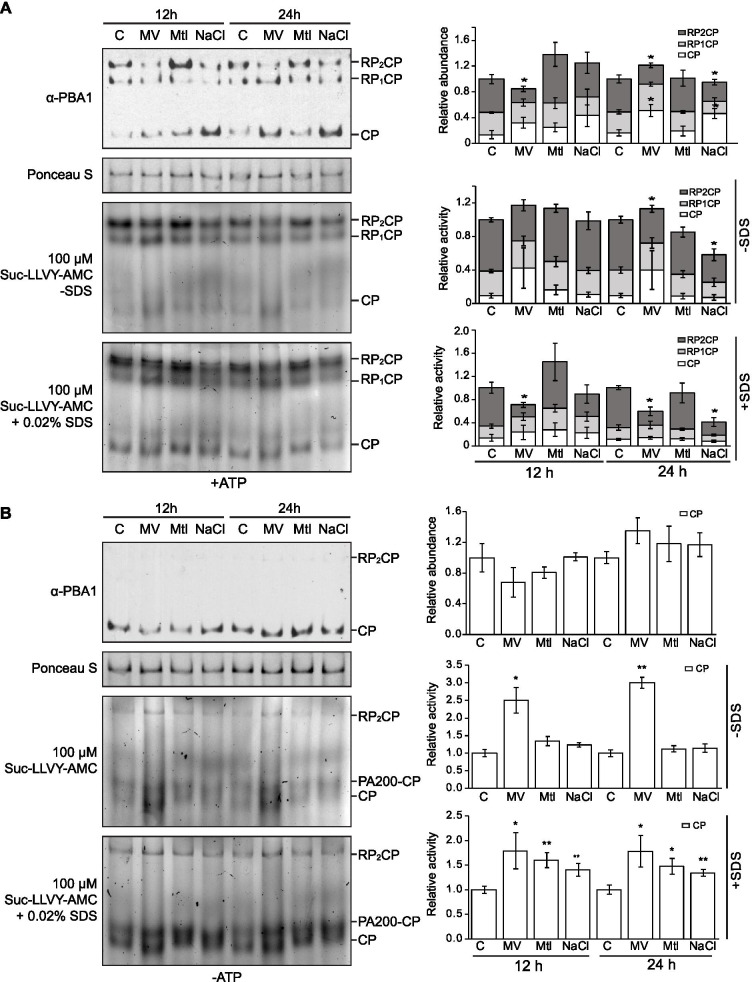
Oxidative and salt stresses change the stoichiometry of proteasome complexes and alter activities of CP. **A**, **B** Anti-PBA1 immunoblot and Suc-LLVY-AMC (100 μM) stain of total lysates resolved on 4% Native-PAGE showing abundance and peptidase activity of the proteasome particles, respectively. *PAG1-FLAG* seedlings were stress-treated for 12 and 24 h before lysis in extraction buffers with (**A**) and without (**B**) 20 mM ATP. Loading was normalized based on fresh weight and a Ponceau S stain is included as a loading control. 0.02% SDS was used to artificially activate the free 20S CP. Three biological replicates were conducted, and one is shown. Bar graphs show digital quantifications of band intensities from immunoblots or activity gels. Data is expressed relative to the control for each time point, which is arbitrarily assigned a total signal value of 1.0. For Native-PAGE run with exogenous ATP, quantifications of RP_2_CP, RP_1_CP, and free CP are expressed as a fraction of total sample signal within stacked bars. All bar charts display the means of three biological replicates ± SEM. Significant differences in RP_2_CP, RP_1_CP or free CP signal between control and stress conditions were determined by independent Student’s t-tests (* = *p* < 0.05, ** = *p* < 0.01). It should be noted that physiological level of ATP is required to maintain the association of RP to CP. In B only trace RP1CP and RP2CP signals were shown in this representative Western blotting image and this particular exposure was chosen to best maintain the free CP signals in linear range

To understand whether the relative amount of different major proteasome ensembles (e.g. RP_2_CP, RP_1_CP and free CP) were affected following exposure to the stressors, we analyzed the proteasome profile isolated in the presence of ATP, which is known to promote association between the 19S and 20S proteasome [42]. Native-PAGE followed by immunoblotting indicated that oxidative and salt stress, but not osmotic stress, caused a reduction in RP_2_CP species with a concurrent increase in the amount of free CP especially after 24 h of stress exposure (Fig. 2A, top panel), without seemingly affecting the total amount of CP particles considering the total PBA1 level (Fig. 1B-C). In-gel peptidase assays with the artificial fluorogenic substrate Suc-LLVY-amc indicated that the relative activities of capped complexes generally matched well with their relative protein amounts (Fig. 2A, middle and bottom panels). Trace amounts of SDS have been widely used to open the α-ring gate and visualize free CP activity in vitro [41]. Interestingly, in the absence of exogenous ATP, the free CP of oxidatively-stressed seedlings already displayed considerable activity before SDS addition and was barely enhanced after SDS addition (Fig. 2B). This suggests that the different stresses exert different effects on the stoichiometry and activity of the proteasome.

The same total cell lysate samples were also subjected to size exclusion chromatography (SEC) followed by immunoblotting. In the presence of ATP, PAG1-FLAG, representing primarily both the CP-containing 20S and 26S proteasomes, was detected in lower molecular weight complexes under salt-stress compared to the control (Supplementary Fig. S2A bottom panel). This suggests increased free 20S CP accumulation under salt stress, in agreement with the Native PAGE (Fig. 2A). However, in oxidatively-stressed samples, PAG1-FLAG did not appear to be shifted much to lower molecular weight complexes (Supplementary Fig. S2A, middle panel). We hypothesize that in the presence of exogeneous ATP, more substrates are processed and associated with CP under oxidative stress, thus giving the CP a higher apparent molecular weight when analyzed by SEC. This is plausible as in the Native gel incubated with Suc-LLVY-AMC, CP bands did appear to migrate slightly faster in oxidatively-stressed samples (Fig. 2A-B), likely due to both altered charge states and sizes of substrate-CP complexes in comparison to free CP. As expected, in samples without exogenous ATP, PAG1-FLAG protein complexes were eluted at very similar fractions in all samples (Supplementary Fig. S2B).

**Fig. 3:**
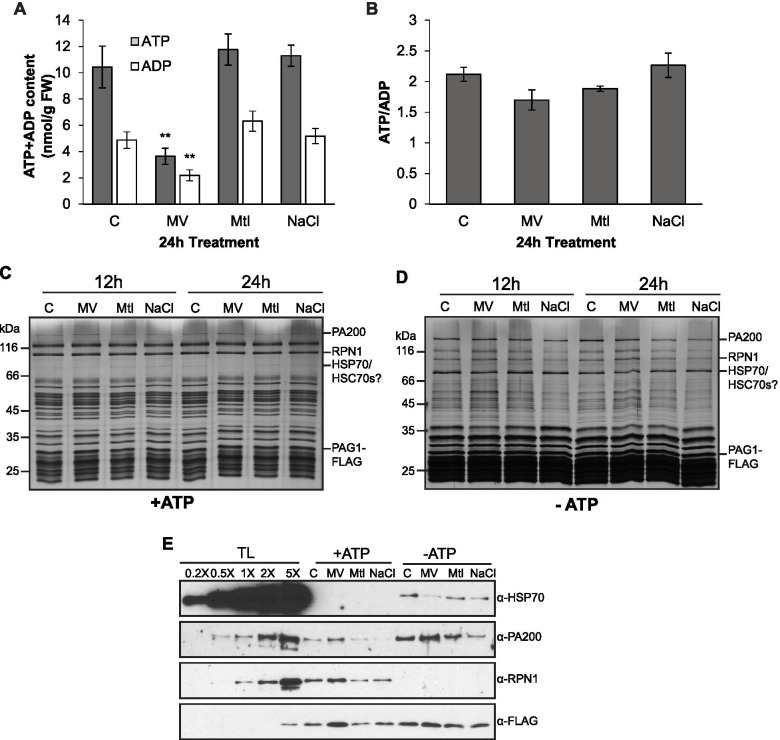
Cellular ATP contents and extrinsic proteasome-associated proteins are affected by oxidative and salt stresses. **A, B** Cellular levels of ATP, ADP (**A**) and ATP/ADP ratio (**B**) of *PAG1-FLAG* seedlings stress-treated for 24 h. Data are displayed as means ± SEM of six biological repeats. Significant differences between control and stress conditions were determined by independent Student’s t-tests (* = *p* < 0.05, ** = *p* < 0.01). **C, D** Co-immunoprecipitation (Co-IP) of PAG1-FLAG-tagged proteasomes from 12- and 24-h stress-treated seedlings via M2 anti-FLAG antibody resin. Cell lysis and co-IP were performed in the presence (**C**) and absence (**D**) of 20 mM ATP. Eluates are visualized on a silver-stained 12% SDS-PAGE gel. **E** Immunoblotting analysis with anti-HSP70, anti-FLAG, anti-RPN1, and anti-PA200 antibodies performed on Co-IP eluates from the 12-h time point shown in (**C, D**). For the immunoblots, Co-IP eluates were loaded alongside increasing concentrations of total lysate (TL) obtained from the 12-h control (C) sample (+ATP)

Cellular stress is generally associated with a reduction in steady state ATP content [3, 36, 67]. The reduction of RP-capped complexes under certain conditions could be correlated with a deficiency in ATP, which is required to maintain the association between RP and CP. To test this premise, the ATP and ADP contents in seedlings that had been oxidatively, osmotically, or salt stressed for 24 h were quantified by a D-luciferin/luciferase assay. Both ATP and ADP levels were significantly lower in MV-treated plants (Fig. 3A), although no real change was observed in the ratio of ATP/ADP (Fig. 3B). Conversely, osmotically- and salt-stressed seedlings did not show any significant deviation from the control treatment in terms of ATP and ADP concentrations or the ATP/ADP ratio. Thus, ATP deficiency may explain the reduction of RP_2_CP under oxidative stress, but a different mechanism is likely to account for RP_2_CP reduction under salt stress.

### Proteasome subunit composition and extrinsic proteasome-associated proteins are affected by abiotic stresses

To understand if abiotic stresses indeed affect the incorporation of different subunits, we purified 20S and 26S proteasomes from stress-treated seedlings by virtue of the FLAG-tagged PAG1 core subunit. Silver staining of the eluates on SDS-PAGE revealed only subtle differences in the protein profile (Fig. 3C-D). These eluates were then subjected to LC-MS/MS analysis. As a result, a total of 190 high fidelity proteins were identified, each with at least two exclusively unique peptides (Supplementary Table S3; see Methods). Out of these proteins, 131 were identified regardless of the presence or absence of ATP (Supplementary Fig. S3A) and included most of the 26S core subunits from which the majority of the uniquely identified peptides were recorded (Supplementary Fig. S3B). Twenty five proteins were identified exclusively in the absence of ATP (Supplementary Fig. S3A, Table S3). On the other hand, 34 proteins were found exclusively in the presence of ATP, although six of them are RP subunits (Supplementary Table S3), thus supporting the important role of ATP in the maintenance of RP-CP association.

As expected, all constituent 26S subunits were identified when exogenous ATP was provided except SEM1/DSS1which was missing in the NaCl-stressed samples (Table 1). Most paralogous pairs (e.g. PAD1/PAD1, PBB1/PBB2 and RPN1a/RPN1b) were identified equally well in all three conditions, except that for PAF, RPT5, RPN2 and RPN5, one paralog was identified with many more peptides than the other. Additionally, no unique peptides were identified for PAC2, RPT1b or RPN12b, suggesting these three subunits may not be well expressed or constitute pseudogenes [7]; this also agrees with the microarray data as previously shown (Fig. 1A). Not surprisingly, most RP subunits were identified in samples purified without exogenous ATP; however, the total unique peptides or the overall protein coverages were much reduced and were only about half of those purified with exogenous ATP (Table 1). This confirms that the physiological level of ATP is a factor that promotes RP-CP association, but is not absolutely required for their association as previously observed [7, 27]. Interestingly, three unique peptides were identified for RPT2a in the presence of exogenous ATP, but only in salt stressed samples, suggesting RPT2a may play a special role in salt stress response.

**Table 1:**
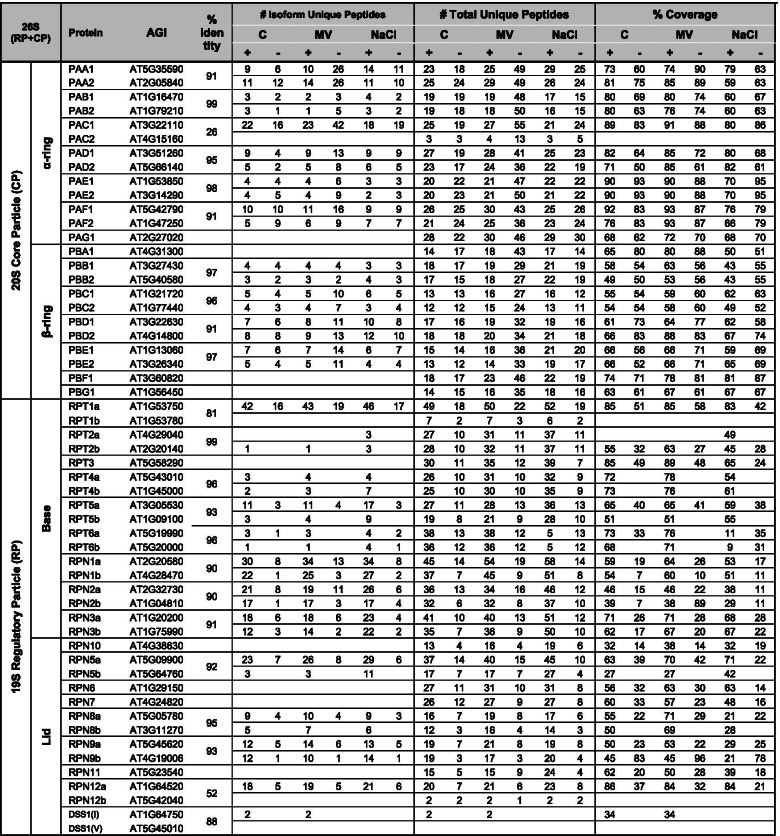
Summary of peptide counts and coverages for core 26S proteasome subunits

Samples purified with and without exogenous ATP were indicated with “+” and “-” respectively. The percentage coverages are analyzed by Scaffold and only shown for subunits that have exclusively unique peptide identified. If a unique peptide was identified in conserved sequence of the two paralogs, it was applied to both even though no exclusively unique peptide was identified for one paralog

Mass spectrometry analyses also detected the presence of several proteasome-associated proteins (PAPs) (Supplementary Fig. S3C, Tables S3 and S4). Interestingly, almost all cytosolic HSP70 proteins accumulated in proteasome preparations where ATP was lacking but were barely detectable when ATP was supplied exogenously. This suggests that HSP70s have a higher affinity for the 20S proteasome than the 26S proteasome. On the other hand, in the absence of ATP, HSP70s were less abundant in MV-treated proteasomes compared to MS and NaCl-treated samples (Supplementary Fig. S3C, Table S4).

The MS analysis was supported by immunoblotting the eluates with anti-HSP70 antibody (Fig. 3E). In contrast, salt stress impacted association of the proteasome with alternative regulators or “caps” (PA200, PTRE1) and CP assembly chaperones (PBAC1-4, PAP1, UMP1a) isolated in the presence and/or absence of ATP (Supplementary Fig. S3C). Reduction of PA200 levels in salt-stressed eluates was also verified by immunoblotting analysis with anti-PA200 antibody, and the efficacy of affinity purification was demonstrated by an immunoblot showing non-detectable RP subunit RPN1 in the purified 20S CP sample when leaving out exogenous ATP (Fig. 3E).

Altogether, these results show that abiotic stresses differentially affect the incorporation of certain proteasome subunits and the binding of regulatory PAPs.

### Mutations in several PAPs negatively impact seedling growth

Our co-IP experiment suggested differential binding of PAPs to the proteasome under stress. PAPs are of interest, as assembly chaperones govern cellular proteasome levels whereas alternative caps modulate proteolytic activity of the 20S CP, which may be important in stress adaptation [24]. Thus, we sought to further examine the biological role of selected PAPs through genetics analysis. We obtained and confirmed several mutants carrying a T-DNA insertion in the exons or 5’UTRs of representative PAP genes (Supplementary Fig. S4; Table S1). In addition to the alternative caps (PA200) and CP assembly chaperones (PBAC1-4, UMP1a/b) that were pulled down with PAG1-FLAG (Supplementary Table S4), we also included assembly chaperones of the RP base (HSM3) and the CDC48 family proteins. CDC48 proteins were predicted to act as alternative caps, but most likely share a transient interaction with the proteasome [5]. Both CP and RP subunits have been caught as *Arabidopsis* CDC48A partners [49], while proteasomal association of CDC48B-D has been implied but not yet fully established [18, 52].

**Fig. 4:**
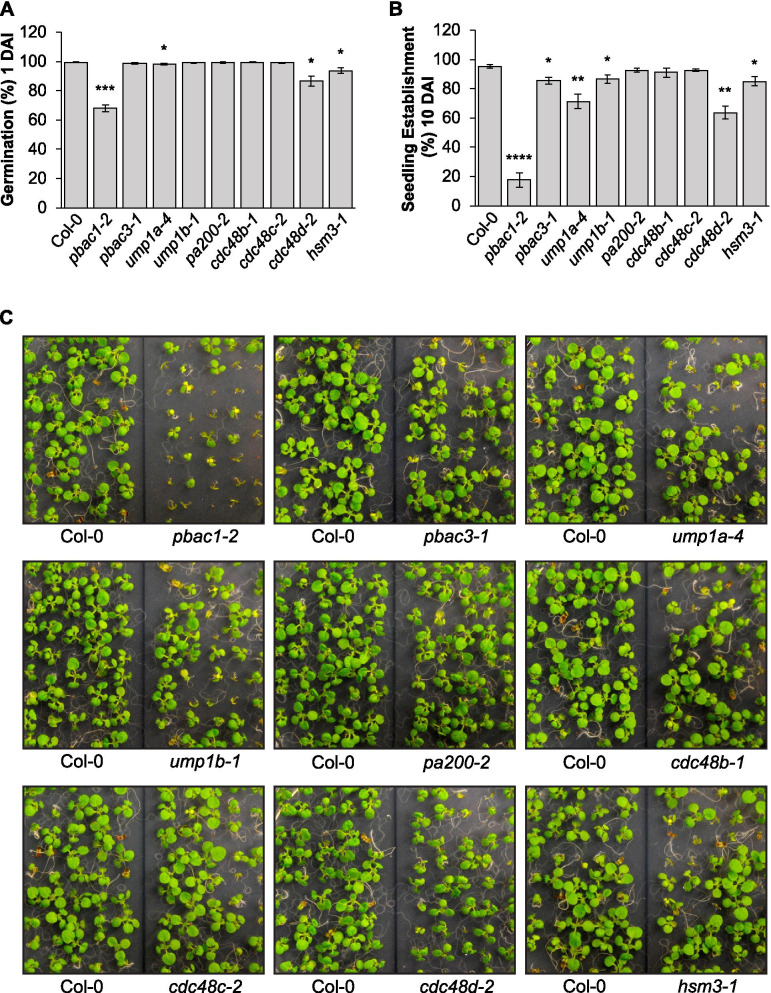
T-DNA insertion mutants in proteasome-associated proteins (PAPs) display arrested or delayed seedling growth. **A, B** Seed germination (radicle protrusion) and seedling establishment (seedlings that showed at least four true leaves) rates were quantified at 1 and 10 days after imbibition (DAI), respectively. Bar charts express the mean ± SEM of five biological repeats (*n* = 124 seedlings). Independent Student’s t-tests were used to assess statistical significance of differences between each mutant and Col-0 (* = *p* < 0.05, ** = *p* < 0.01, **** = *p* < 0.001). **C** Images of Col-0 and homozygous *pbac1-2*, *pbac3-1*, *ump1a-4*, *ump1b-1*, *pa200-2*, *cdc48b-1*, *cdc48c-2*, *cdc48d-2*, and *hsm3-1* mutants grown on half-strength MS media for 10 DAI

Under optimal conditions, young seedlings of some mutant lines showed non-uniform growth, with some seedlings germinating but being visibly arrested soon after germination (Fig. 4A-C), suggesting that PAPs may play a role in regulating seedling growth after emergence from the seed coat. Out of all tested mutant lines, *pbac1-2* seeds showed the most severe germination and growth defects. *ump1a-4* and *cdc48d-2* mutants also showed significant, albeit less severe growth defect, while the *pa200-2* mutant did not show any difference from wild type, in agreement with a previous study [27] .

**Fig. 5:**
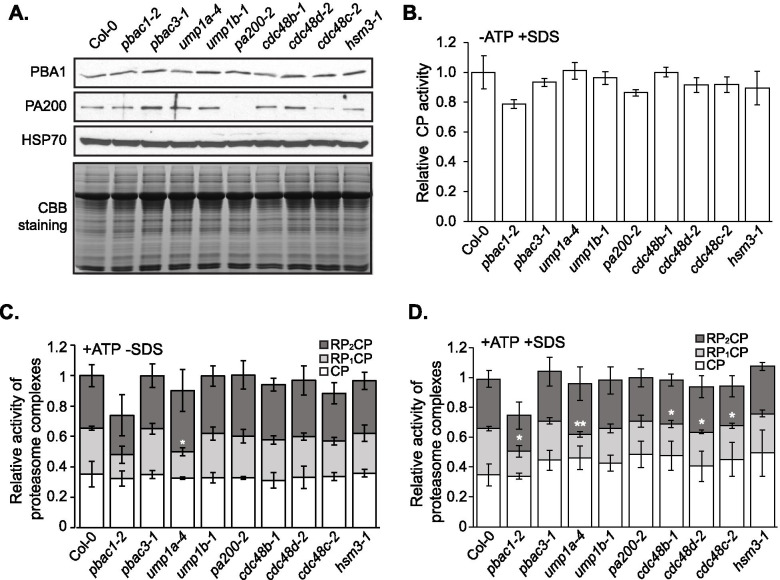
Decreased activity of the RP_1_CP species in proteasome-associated protein (PAP) mutants. *pbac1-2*, *pbac3-1*, *ump1a-4*, *ump1b-1*, *pa200-2*, *cdc48b-1*, *cdc48c-2*, *cdc48d-2*, and *hsm3-1* mutants were grown for 10 days on half-strength MS media as shown in Fig. 4C. **A** Anti-PBA1, anti-PA200, and anti-HSP70 immunoblots of PAP mutant lysates resolved on 12% SDS-PAGE. **B** Quantitative analysis of Suc-LLVY-AMC (100 μM) stain signals of total lysates prepared and resolved on 4% Native-PAGE without any exogenous ATP. **C**, **D** Quantitative analysis of Suc-LLVY-AMC (100 μM) stain signals of total lysates prepared with 20 mM ATP and resolved on 4% Native-PAGE with 1 mM ATP. The native gels were stained and quantitated without (**C**) or with (**D**) incubation of 0.02% SDS to artificially activate the 20S CP. All mutant signals are expressed relative to that of Col-0, which is arbitrarily assigned a total signal value of 1.0. For gels run with exogenous ATP, quantifications of RP_2_CP, RP_1_CP, and free CP are expressed as a fraction of total sample signal within stacked bars. All bar charts display the means of three biological replicates ± SEM. Significant differences in RP_2_CP, RP_1_CP or free CP signal between each mutant and Col-0 were determined by independent Student’s t-tests (* = *p* < 0.05, ** = *p* < 0.01)

To determine whether mutations in PAP genes could affect the levels of proteasome subunits, lysates from 10-day-old mutant seedlings were resolved on SDS-PAGE and immunoblotted with anti-PBA1 antibody (Fig. 5A). Steady-state levels of PBA1 were relatively consistent across all lines. An immunoblot with anti-PA200 antibody confirmed the lack of this protein in *pa200-2* (Fig. 5A). To further examine the role of PAPs in proteasome complex formation, seedling lysates were resolved on Native-PAGE with and without exogenous ATP, and the relative activity of different proteasome ensembles were analyzed with fluorogenic substrate Suc-LLVY-amc. The CP activities in the presence of the artificial activator SDS were not significantly different between any PAP mutant and the wild type seedlings (Fig. 5B, D), either with or without ATP suggesting that no single PAP is essential to 20S CP proteasome complex formation in established seedlings. However, with exogenous ATP, we observed a greatly reduced activity of the RP_1_CP in *pbac1-2* and *ump1a-1*, as well as somewhat reduced RP_1_CP activity in the three selected *cdc48* mutants (Fig. 5C, D). This suggests that the observed growth defects of these mutants might be due to reduced proteasome activity.

### Increased sensitivity of PAP mutants to oxidative and/or salt stress

**Fig. 6:**
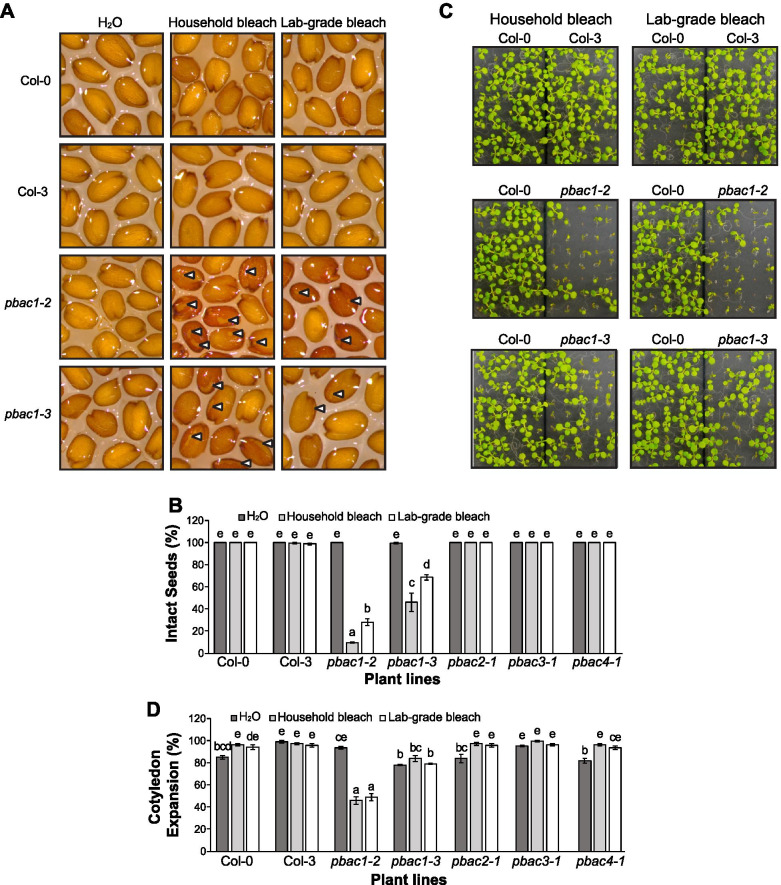
Darkening and tearing of *pbac1* seed coat following sterilization with bleach. Dry seeds were either imbibed in H_2_O (non-sterile), household bleach (LAVO-12; 2.58% NaOCl), or laboratory-grade bleach (BioShop; 2.58% NaOCl). **A** Enlarged images of Col-0, Col-3, *pbac1-2* and *pbac1-3* seeds following treatment. Holes in the seed coat are indicated by white arrowheads. **B** Proportion of treated seeds without visible holes, namely intact seeds (**C**) Images of Col-0, Col-3, *pbac1-2*, and *pbac1-3* seeds that had been treated with household or lab-grade bleach and then grown on half-strength MS media for 10 DAI. Note that non-sterile seeds could not be grown to this stage due to contamination. **D** Proportion of treated seedlings with expanded cotyledons at 3 DAI. Bar charts display the means of three biological repeats (*n* = 50 seeds and *n* = 56 seedlings, respectively) ± SEM. Means that do not share a common letter differ significantly (*p* < 0.05) as analyzed by two-way ANOVA and Tukey’s HSD test. Note that *pbac1-2* and *pbac1-3* mutations are in the Col-3 ecotype. The remainder of the mutants are in the Col-0 ecotype

During our preliminary screen of PAP mutants, *pbac1-2* exhibited the strongest physiological phenotype, which correlated with decreased proteasome activity. Upon closer inspection, we observed that after surface sterilization with either household bleach or laboratory grade sodium hypochlorite (NaOCl) solution, *pbac1-2* seed coats were visibly darkened and bore small holes in the testa, whereas non-treated *pbac1-2* seeds were identical to WT (Fig. 6A). To confirm this phenotype, we obtained a second mutant allele (*pbac1-3*), as well as mutants of other genes within the same family (*pbac2-1*, *pbac3-1*, *pbac4-1*) (Supplementary Fig. S4). Darkening and tearing of the seed coat were reproduced in *pbac1-3*, albeit to a milder extent (Fig. 6AB). A significant number of *pbac1-3* seedlings also displayed growth defect (Fig. 6CD). Conversely, no other *pbac* mutant was negatively affected by bleach-based sterilization (Supplementary Fig. S5). Given that *pbac1-2* and *pbac1-3* mutants were isolated in the Col-3 background (Table S1), we also tested the seed phenotype, germination, and seedling growth of Col-3. However, no phenotypic or growth differences were seen between Col-0 and Col-3 (Fig. 6).

NaOCl is a strong oxidant and has been widely used for surface sterilization of seeds. We reasoned that other oxidizing agents, such as hydrogen peroxide (H_2_O_2_) and MV might reproduce the *pbac1-2* phenotype. Surprisingly, out of all agents tested, NaOCl was the only chemical that induced signature darkening and tearing of the *pbac1-2* testa (Supplementary Fig. S6). Triton X-100 enhanced this effect in combination with NaOCl but could not produce any seed coat damage on its own. 10% H_2_O_2_ caused substantial shriveling of the testa, but this effect was observed equally in both wild type and *pbac1-2*. Oxidants like 10% H_2_O_2_ or 2 μM MV were capable of compromising seed germination in WT as well as *pbac1-2* (Supplementary Fig. S7). This suggests that the strong growth defects of *pbac1-2* seedlings are specifically caused by the NaOCl.

**Fig. 7:**
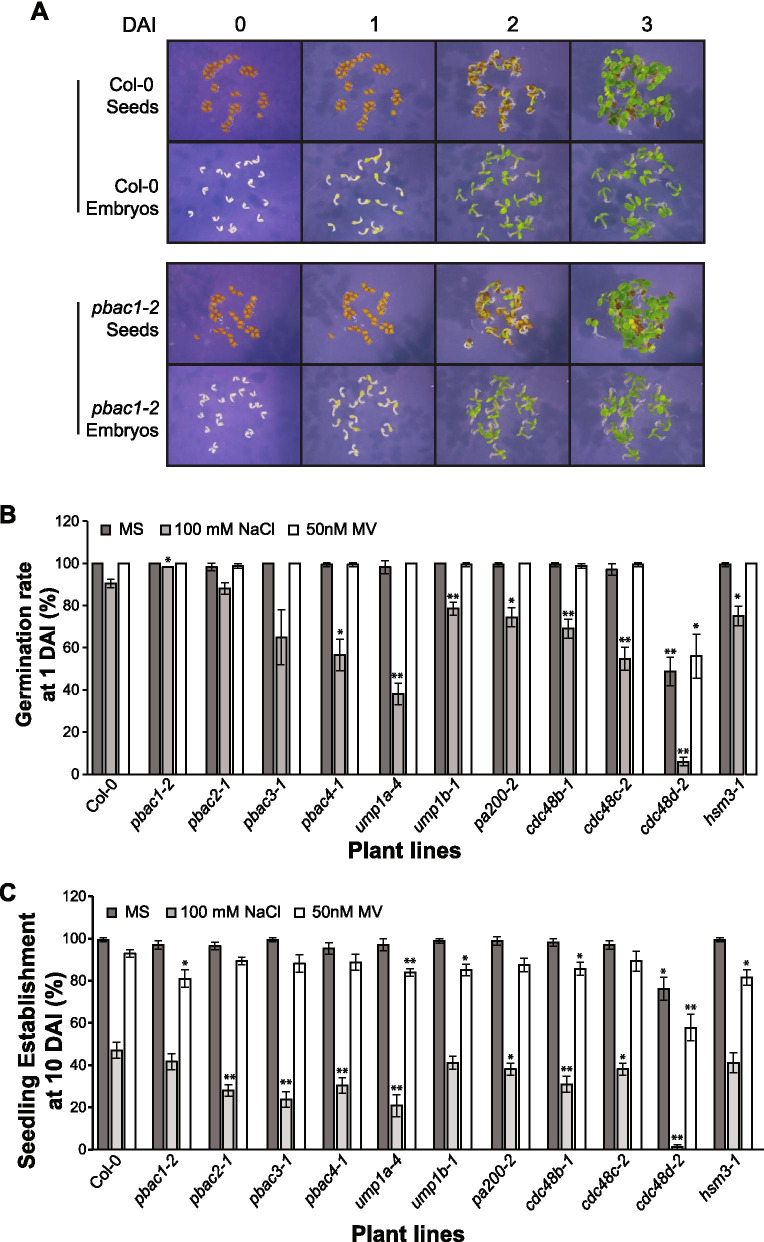
Germination and seedling development of PAP mutants under salinity and oxidative stresses without bleach sterilization. **A** Images of non-sterile whole seeds or dissected embryos from Col-0 and *pbac1-2* lines sown on half-strength MS media from 0 to 3 DAI. **B, C** Germination rate at 1 DAI (**B**) and seedling establishment at 10 DAI (**C**) of wild type (Col-0) and PAP mutant seeds on half-strength MS media (MS), 100 mM NaCl or 50 μM MV. Bar charts display the means of three biological repeats (*n* = 56 seeds and *n* = 56 seedlings, respectively) ± SD. Student’s t-test with two-tailed distribution and two-sample unequal variance was performed for each mutant line and the Col-0 was used as reference. * = *p* < 0.05 and ** = *p* < 0.01, respectively

To better understand the natural germination and development process of the *pbac1* mutants, we plated the intact seeds and embryos that were dissected from their seed coats directly on MS media without prior bleach sterilization. Interestingly, the growth of *pbac1-2* seedlings was nearly identical to wild type in both cases (Fig. 7A). This suggests that bleach sterilization may specifically damage the *pbac1-2* embryos after penetrating the seed coat (Figs. 4 and 6).

To overcome the negative impact of bleach, and to further assess the role of PAP genes in stress response during the early developmental stages, we developed a procedure to sterilize seeds only with 70% ethanol. Not surprisingly, except for *cdc48d-2*, all other PAP mutants including *pbac1-2*, *pbac2-1*, *pbac3-1* and *pbac4-1* reached nearly 100% germination (Fig. 7B) and seedling establishment (Fig. 7C) on MS media like wild type. When grown on MV media, six PAP mutants *pbac1-2*, *ump1a-4*, *ump1b-1*, *cdc48b-1*, *cdc48d-2* and *hsm3-1* showed increased sensitivity and defective seedling establishment at 10 DAI compared to wild type, though their germination was not significantly affected. In contrast, germination (Fig. 7B) or seedling establishment (Fig. 7C) of all PAP mutants was significantly affected by salinity stress. This is in line with RNA expression analysis showing these PAP genes are differently regulated under salt or oxidative stresses (Supplementary Figure S8).

Overall, our data indicate that the proteasome assembly chaperone or regulator mutants exhibit different sensitivity to salt and/or oxidative stresses, suggesting that these genes play an important role in response to oxidative and/or salinity stresses during seed germination and seedling establishment.

## Discussion

**Fig. 8:**
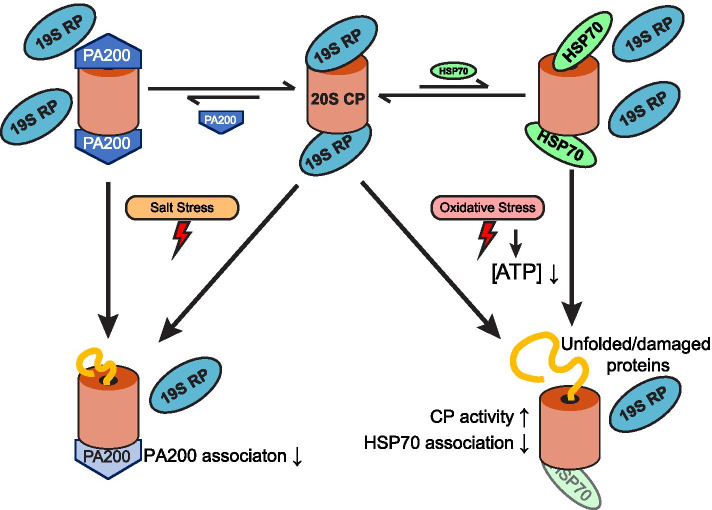
Model of proteasome dynamics under oxidative and salt stresses. Cellular 26S proteasome is dynamically assembled and disassembled from 19S RP and 20S CP, and associates with chaperones such as HSP70, as well as alternate caps, including PA200. Oxidative and salt stresses both diminish the association between the 19S RP and the 20S CP, and oxidative stress may trigger 26S proteasome dissociation indirectly through a reduction in cellular ATP content. Activity of the dissociated or free 20S proteasome is increased under oxidative stress, possibly due to its greater propensity for the open state or higher affinity to oxidized and misfolded proteins. While the 20S proteasome also exhibits reduced association with HSP70s under oxidative stress, salt stress causes decreased interaction with proteasome associated proteins, such as PA200. Note that the proteasome particles and associated proteins are not drawn in scale

The regulation of proteasome function and structure during stress has been well explored in yeast and human cells [1, 4, 24, 54] and has drawn attention also in plant cells in recent years [47, 77]. Proteasome genes were shown to be upregulated at the transcriptional level upon proteotoxic stresses, e.g. treatment of proteasome inhibitor MG132, and environmental factors, likely through the control of a cohort of transcription factors including NAC53 and NAC78 [28]; Fig. 1A). Our analysis of proteasome complex architecture during abiotic stress reveals an evolutionarily conserved mechanism of proteasome regulation under ATP-limiting conditions, which occurs during oxidative stress. This includes the dissociation of the 26S proteasome, a subsequent increase in the abundance and activity of the CP, as well as reduced HSP70 association with the CP. HSP70 has been previously shown to mediate proteasome dissociation and reassociation under oxidative stress in human cells [31] and our study suggests this chaperone also plays a role in proteasome dynamics in plants. Although salt stress did not affect cellular ATP levels, or the association of HSP70 to either 26S or 20S proteasome, it negatively affected the association of PA200 to the proteasome, suggesting differential regulation of proteasome architecture and function under salt and oxidative stresses. Last, our data highlights a role for PAPs in regulating the transition from seed to seedling, and specifically a function for PBAC1 in preventing oxidative damage of the seed coat. Taken together, we propose a model (Fig. 8) in which energy (ATP) levels and proteasome-associated heat-shock or assembly chaperones might assist proteasome remodeling to better respond to abiotic stresses such as oxidative and salt stresses.

### Re-modeling of the 26S and 20S proteasome under abiotic stress

The 20S proteasome is particularly relevant to oxidative stress, not only because oxidized proteins are the major substrates, but also because the 20S complex can withstand more oxidative modification than the 26S before experiencing appreciable decreases in peptidase activity [57, 58]. In the present study, oxidative stress was found to reduce the levels of 26S proteasomes with a concurrent increase in the amount of free 20S, which is by itself latent, but can be activated by many reagents including hydrophobic groups or disordered segment of proteins [2, 19]. This change between 26S and 20S, coupled with the lack of a significant change in steady-state levels of RPN1, PBA1, PAG1-FLAG, and likely fully assembled total CP, implied that the 26S proteasome was dissociating into its constituent subcomplexes under these conditions, as was previously reported in yeast and mammals [31, 73, 74]. This evolutionarily conserved response seems to be a means to immediately boost 20S proteasome levels, without involving transcription and translation of new CP subunits. Interestingly, we observed a similar pattern for 26S dissociation under salt stress. Given that abiotic stresses generally lead to the build-up of ROS, though ROS accumulation may need different stress treatment periods and intensity [17], it is possible that both MV and NaCl treatments eventually resulted in the oxidation of cellular components and thus induced a similar form of structural adaptation in the proteasome.

Normally, the isolated 20S proteasome is latent due to the N-terminal tails of α-subunits preventing substrate entry and subsequent degradation [29, 44]. Here, we found enhanced activity of the endogenous 20S proteasomes under oxidative stress even before partial denaturation using SDS. This suggests that oxidative stress increases substrate access to β-subunit catalytic sites, possibly via α-ring gate opening. In support, prior yeast studies have shown that gate-opening can be triggered by modifications of the 20S proteasome such as *S-*glutathionylation, which are more likely to occur in an oxidative environment [40, 62]. This response is likely a means of accelerating degradation of oxidized proteins once the 26S proteasome has dissociated, the cellular proportion of 20S is high, or the 19S RP is no longer available to mediate substrate targeting. Enhanced substrate accessibility may ultimately have a cell-protective role, as open-gate mutations (α3ΔN) were shown to improve the viability of mammalian cells exposed to ROS-inducing agents [15].

### Altered proteasome subunit incorporation and association of interactors under abiotic stress

LC-MS/MS analysis revealed altered association of several proteasomal subunits and PAPs in the salt-stressed proteasome. Under salt stress, PAG1-FLAG pulled down less of the alternative caps, PA200 and PTRE1, along with less of the CP assembly chaperones, PBAC1-4, PAP1, and UMP1a. Assembly chaperones primarily bind proteasome intermediates and dissociate or are degraded once proteasomes fully mature [10]. Thus, the co-purification of fewer assembly chaperones under salt stress suggests the presence of fewer proteasome intermediates or a possibility that the assembly process has been somehow altered.

HSP70-family proteins co-purified with CP-tagged proteasomes mostly in ATP-limiting conditions, wherein the RP-CP association was perturbed. This is consistent with the hypothesis that HSP70 takes over as a regulator of substrate targeting to the 20S proteasome when the 19S is no longer available for binding in yeast cells [56]. However, under oxidative stress, the interaction between HSP70s and 20S proteasomes was dampened possibly due to increased recruitment of HSP70 by oxidized proteins. It is worth mentioning here that the effect of HSP70 on proteasome function may be multi-faceted. Under normal conditions, human HSP70 was found to activate the 26S while inhibiting the 20S proteasomal degradation of artificial fluorogenic peptides [51]. However, during short exposure (1-3 h) to mild oxidative stress, HSP70 recruited 19S regulators to release more 20S CP that could be subsequently activated by other activators in human cells [31]. Furthermore, during recovery stage after oxidative stress, HSP70 is upregulated and enhances the degradation of oxidized proteins by the 20S proteasome in mouse cells [56]. Clearly, the role of HSP70 in proteasomal degradation is hard to pinpoint because it depends on cellular context, the type and duration of stress, as well as the substrates that are being engaged. Thus, further investigation in the same context is required.

### Role of proteasome-associated proteins (PAPs) in proteasome function and plant fitness

Among the PAPs, proteasome assembly chaperones and alternate caps are vital to the generation of new proteasomes and activity regulation within the 20S core. These proteins exhibited differential binding to the proteasome under stress. Overall, T-DNA insertions in *pbac1-2*, *ump1a-4*, and *cdc48d-2* caused a strong growth delay or arrest. While these mutations were not associated with decreased abundance of proteasomes, they did result in reduced peptidase activity of the RP_1_CP species, suggesting a portion of proteasomes may be assembled aberrantly. This is partly supported by a recent study showing that multiple proteasome assembly intermediates, including free CP β-subunits and incomplete α-rings, were observed when UMP1a was absent [27]. The same study also showed that *ump1a-2* and *ump1b-2* mutants were hypersensitive to NaCl and mannitol. Our findings further support a role for the assembly chaperones of the CP, UMP1a, in regulating proteasome activity, which is required for proper seedling establishment and response to salinity stress, and also suggest a role for other assembly chaperones – PBAC, PA200, CDC48 and HSM3 – in seed germination and/or seedling establishment under mild salinity and/or oxidative stress. This is in agreement with the up- or down-regulation of most PAP genes observed under these stresses. Interestingly, among the five CDC48 family members, only CDC48A has been shown to play a role in immune responses [18]. Our study shows a prominent role for CDC48D in salt stress, suggesting functional diversification of CDC48 family members. Furthermore, we also show that PBAC1-PBAC4 are required for proper response to salinity stress, although PBAC2, PBAC3 and PBAC4 seem to play a more prominent role at least at the NaCl concentration tested (100 mM). Our observation agrees with a recent report that showed hypersensitivity of T-DNA insertional mutants for PBAC1, PBAC2 and also PBAC5 to 50 mM NaCl [45]. Although all *pap* mutants showed only a mild sensitivity to oxidative stress, this may be due to the low concentration of MV used in these assays.

A recent study in maize suggested that the PBAC family of α-ring assembly chaperones might be particularly important in seed maturation, as a loss of function mutation in PBAC4 (*dek40*) retarded embryo and endosperm development, resulting in collapsed kernels [71]. Additionally, the positive role of the ubiquitin proteasome system in promoting germination of Arabidopsis and hybrid wheat seeds has been reported [14, 78]. Our analysis agrees with these studies, as *pbac1-2* and *pbac1-3* displayed significant germination and growth defects, in particular when treated with the strong oxidant NaOCl that could specifically oxidize the testa and over-stress the embryos in this mutant line. This is particularly interesting because ROS accumulation is known to play a role in seed development and dormancy breaking, and over accumulation of ROS could also lead to oxidative damage of both mature dry and imbibed seeds [23, 35]. Since PBAC1 is expected to assist in 20S proteasome assembly, which is pivotal in degrading oxidized proteins, the growth defect of *pbac1-2* seedlings could be partly due to its inability to clear those proteins being damaged by ROS during the early growth stage. However, we cannot rule out other possibilities. For instance, bleach may better penetrate the seed coat and oxidize some specifically accumulated proanthocyanidins (PAs) in the *pbac1-2* line, which are normally shielded by the mucilage and secondary cell wall of the epidermis [34], thus causing the darkening of the seed coat.

## Conclusions

In conclusion, this study provides evidence for differential regulation of proteasome holoenzyme modular assembly during the salinity and oxidative stresses in Arabidopsis. Particularly, we identified a prominent role of multiple PAPs in regulating seed-to-seedling transition, and a specific role for PBAC1 in modulating seed coat development and sensitivity to oxidative stress, thus broadening our understanding of complex and regulated cellular protein degradation mechanism by the 26S proteasome.

## Methods

### Plant materials, growth conditions and stress assays

T-DNA insertion mutant seed stocks were obtained from the Arabidopsis Biological Resource Center (ABRC; https://abrc.osu.edu/) and are listed in Supplementary Table S1. Mutants were selected on 25 μg/mL kanamycin, 20 μg/mL glufosinate ammonium (BASTA) or 7.5 μg/mL sulfadiazine. Resistant seedlings were transferred to soil and the presence of the T-DNA insertion was confirmed by PCR with primers listed in Supplementary Table S2. All seeds were surface sterilized with bleach solution (2.58% NaOCl, 0.1% Triton X-100) unless specifically indicated. In our bleach sterilization test, two sources of bleach were used, one labelled as household bleach that was purchased as general disinfectant (LAVO-12™, 12% sodium hypochlorite) and the other labelled as lab-grade bleach which was purchased from BioShop (sodium hypochlorite, SYP001, 12% solution). For abiotic stress treatments, *pag1-1 pPAG1*:*PAG1-FLAG* (Col-0 background) seeds, which were originally provided by Richard Marshall (Washington University) and previously described [7], were sown on half-strength Murashige-Skoog (MS) media (pH 5.6/KOH), containing 1% sucrose and 0.4% agar. Stress treatment procedure was adapted based on a previous study [37]. Briefly, eight-day-old *PAG1-FLAG* seedlings were transferred to liquid media (half-strength MS, 1% sucrose, and 0.1% agar) for a 48-h acclimation period. Subsequently, oxidative, osmotic, and salt stresses were induced by supplementing the liquid media with 10 μM methyl viologen (MV), 300 mM mannitol, and 150 mM NaCl, respectively. Seedlings were harvested 12 and 24 h after the onset of stress treatment.

For germination assays and other phenotypic analyses, seeds from all lines (Supplementary Table S1) were sown on half-strength MS media containing 5 mM 2-(N-morpholino)-ethanesulphonic acid (MES; pH 6.0) and 0.8% agar. All seeds were stratified in the dark at 4 °C for 3–5 days and then incubated at 22 °C with 120 μmol·m^− 2^·s^− 1^ and a 16−/8-h light/dark cycle. When propagating lines to the next generation, 7 to 10-day-old seedlings were transferred to soil and grown in a plant growth chamber at 22 °C with 110 μmol·m^− 2^·s^− 1^ and a 16 h/8 h light/dark cycle. Seeds were collected 4 weeks after the completion of plant senescence.

### Affinity purification of proteasomes

The procedures to purify the 26S or 20S proteasomes were adapted from Marshall et al. [46]. Briefly, fresh tissue was ground in liquid nitrogen, then resuspended in 1.25X fresh weight (FW) volumes of extraction buffer A [50 mM 4-(2-hydroxyethyl)-1-piperazineethanesulfonic acid (HEPES)-KOH, pH 7.5, 50 mM NaCl, 10% glycerol, 10 mM MgCl_2_, 5 mM dithiothreitol (DTT), 2 mM phenylmethylsulfonyl fluoride (PMSF), 2 μM chymostatin] either with or without 20 mM ATP for 20 min. The lysate was clarified by centrifugation at 30,000 x g for 30 min at 4 °C and then incubated with pre-equilibrated anti-FLAG® M2 resin (Sigma; A2220). The suspension was incubated for 1 h at 4 °C on a rotary shaker. After washing four times using buffer A with or without 20 mM ATP, bound proteins were eluted using buffer A containing 500 ng/μL 3X FLAG® Peptide (Sigma; F4799). The eluants were subjected to SDS-PAGE and immunoblotting or sent for liquid chromatography coupled with tandem mass spectrometry (LC-MS/MS) at the SPARC BioCentre at SickKids Hospital (Toronto, Canada). Scaffold 4.8.8 (Proteome Software Inc.) was used to refine peptide and protein identifications. Proteins with over 95.0% probability and at least two exclusive unique peptides from at least one sample were accepted for further examination.

### Native- and SDS-PAGE and immunoblotting

Samples destined for Native-PAGE analysis were combined with native sample buffer (50 mM Tris-HCl, pH 7.4, 10% glycerol, 0.0014% xylene cyanol at final concentration) and loaded onto polyacrylamide gels [90 mM Tris-borate, pH 8.35, 5 mM MgCl_2_, 0.5 mM ethylenediaminetetraacetic acid (EDTA), 2.5% sucrose] with 3% stacking and 4% resolving portions. Electrophoresis was performed with native running buffer (90 mM Tris-borate, pH 8.35, 5 mM MgCl_2_, 0.5 mM EDTA) at 100 V for 3 h on ice. Samples prepared with 20 mM ATP were separated using gels and running buffer containing 1 mM ATP. Samples intended for SDS-PAGE analysis were combined with Laemmli sample buffer [39] and resolved on 12% gels. Where necessary, gels were stained with Coomassie Brilliant Blue R-250 or silver nitrate [13]. Alternatively, proteins resolved by Native-PAGE or SDS-PAGE were transferred onto nitrocellulose membrane (PALL BioTrace™; 66,485) and immunoblotted with specific antibodies. Antibodies used in this study include anti-FLAG (Sigma; F1804), anti-PBA1 (Abcam; ab98861), anti-RPN1 (Abcam; ab98865), anti-HSP70 (Agrisera; AS08 371), anti-PA200 (Agrisera; AS19 4269), goat anti-mouse conjugated to horseradish peroxidase (HRP) (Cell Signaling; 7076S), and goat anti-rabbit conjugated to HRP (Sigma; A9169). Chemiluminescent signals were quantified using ImageJ (https://imagej.nih.gov/ij).

### In-gel peptidase activity assay

Proteasome activity was assessed in-gel as described by Li et al. [41]. After electrophoresis, Native gels were quickly rinsed in assay buffer (25 mM Tris-HCl, pH 7.4, 10% glycerol, 10 mM MgCl_2_, 1 mM DTT). If analyzing samples extracted in the presence of 20 mM ATP, 1 mM ATP was added to the assay buffer before use. Gels were then developed in assay buffer containing 100 μM Suc-Leu-Leu-Val-Tyr-7-amino-4-methylcoumarin (Suc-LLVY-AMC; Boston Biochem; S-280) at 30 °C for 30 min. An ultraviolet (UV) transilluminator (Syngene D) emitting light at 365 nm was used to visualize fluorescent signal produced by capped CP. To assess free CP activity, 0.02% SDS was added to the developing solution and the gels were re-incubated at 30 °C for 30 min, followed by UV visualization. The fluorescent signals were quantified using ImageJ.

### Determination of cellular ATP and ADP

Nucleotide extraction procedure was adapted from Ford and Leach [26]. Briefly, stress-treated seedlings were ground in liquid nitrogen, then resuspended in 2.3% trichloroacetic acid and kept on ice for 30 min. After 20,000 x g centrifugation at 4 °C for 15 min, supernatants were neutralized with 1 M Tris-acetate buffer (pH 7.75) until a pH of 6.5–7.0 was obtained. Activated charcoal was added to 0.65% and the resulting suspensions were allowed 20 min on ice for adsorption of coloured compounds. Charcoal was removed by 20,000 x g centrifugation for 10 min at 4 °C, then each clarified nucleotide extract was diluted 100-fold with H_2_O before ATP content determination using the Promega ENLITEN® ATP Assay System (FF2000). To measure the ADP content, ADP was first converted to ATP with 0.1 mM phosphoenolpyruvate (PEP) and 0.08 μg/μL pyruvate kinase (PK) and ADP amounts were indirectly determined by subtraction of ATP from total ATP + ADP.

### Phenotypic characterization of PAP mutants

Various traits of PAP mutant seedlings were scored every day up to 10 DAI, including seed germination (seeds having ruptured endosperm with an emerged radicle), cotyledon expansion (seedlings having cotyledons at least 180^o^ apart), root emergence (seedlings with roots at least 1 mm in length), true leaf emergence (seedlings with at least two rosette leaves with visible trichomes), and seedling establishment (seedlings developing at least four rosette leaves more than 1 mm in length). One hundred and twenty-four seedlings were scored for each developmental trait per line. Also, at 10 DAI, 50 seedlings per line were harvested for FW measurements. The same seedlings were then allowed to stand at 80 °C for 24 h before taking dry weight (DW) measurements. The quantification of photosynthetic pigment content was performed with 20 seedlings per line harvested at 10 DAI and the total pigment contents were calculated as previously described [9].

### Statistical analyses

Two-tailed, unpaired Student’s t-tests assuming unequal variance were used to check for significance (*p* < 0.05) in the comparison of control and treatment group. For experiments having two or more control groups, two-way analysis of variance (ANOVA) assuming interaction between factors (i.e. genotype and chemical treatment) and Tukey’s honestly significant difference (HSD) tests were used to check for significance (*p* < 0.05) in multiple comparisons of means. In the case of protein band quantifications, where all experimental values were normalized to the appropriate control, two-tailed, one-sample t-tests (*p* < 0.05) were conducted assuming a hypothetical mean of 1.0.

## Supplementary Information

**Additional file 1: Table 1.** List of T-DNA insertion mutant lines used in this study.

**Additional file 2: Table 2**. List of primers used in this study.

**Additional file 3: Table 3.** Raw mass spectrometry data for all identified proteins.

**Additional file 4: Table 4.** Short summary of proteasome associated regulator proteins and molecular chaperones.

**Additional file 5: Fig. S1.** Oxidative, osmotic, and salt stress treatment of *PAG1-FLAG* seedlings up to 24 h. **Fig. S2.** Size exclusion chromatography (SEC) analysis of total cell lysates from seedlings. Grown on non-stress (MS), methyl viologen (MV) or salt (NaCl) stressed media. **Fig. S3.** Summary of identified proteins and unique peptides by LC-MS/MS analysis. **Fig. S4.** Confirmation of T-DNA insertion homozygosity within proteasome-associated protein (PAP) mutants. **Fig. S5.** Appearance of all *pbac* mutant seeds, and their subsequent development after treatment with bleach. **Fig. S6.** Effect of various chemical pre-treatments on the appearance of *pbac1-2* seeds. **Fig. S7.** Effect of various chemical pre-treatments on *pbac1-2* seed germination and seedling growth. **Fig. S8.** Effect of abiotic stresses on expression level of PAP genes.

**Additional file 6.**

## Data Availability

The datasets used and/or analyzed are available from the public open database http://bar.utoronto.ca and https://abrc.osu.edu, and newly generated mass spectrometry data are provided in supplemental tables. PAP mutant lines are available from the corresponding author on reasonable request.

## Abbreviations

RP: Proteasome regulatory particle
CP: Proteasome core particle
UPS: Ubiquitin proteasome system
PAP: Proteasome associated proteins
MV: Methyl viologen
LLVY-AMC: Suc-Leu-Leu-Val-Tyr-7-amino-4-methylcoumarin
ROS: Reactive oxygen species
RP_1_CP: Singly capped proteasome
RP_2_CP: Doubly capped proteasome
NaCl: Sodium chloride
