# Supplementary material for: Oxidative and salt stresses alter the 26S proteasome holoenzyme and associated protein profiles in Arabidopsis thaliana
Source: BMC Plant Biol. 2021 Oct 25;21:486. doi: 10.1186/s12870-021-03234-9 (PMC8543921; doi:10.1186/s12870-021-03234-9)
Supplement: Supplementary file 5 — Additional file 5: Fig. S1. Oxidative, osmotic, and salt stress treatment of PAG1-FLAG seedlings up to 24 h. Fig. S2. Size exclusion chromatography (SEC) analysis of total cell lysates from seedlings. Grown on non-stress (MS), methyl viologen (MV) or salt (NaCl) stressed media. Fig. S3. Summary of identified proteins and unique peptides by LC-MS/MS analysis. Fig. S4. Confirmation of T-DNA insertion homozygosity within proteasome-associated protein (PAP) mutants. Fig. S5. Appearance of all pbac mutant seeds, and their subsequent development after treatment with bleach. Fig. S6. Effect of various chemical pre-treatments on the appearance of pbac1-2 seeds. Fig. S7. Effect of various chemical pre-treatments on pbac1-2 seed germination and seedling growth. Fig. S8. Effect of abiotic stresses on expression level of PAP genes. [file 12870_2021_3234_MOESM5_ESM.pdf]

Figure S1.

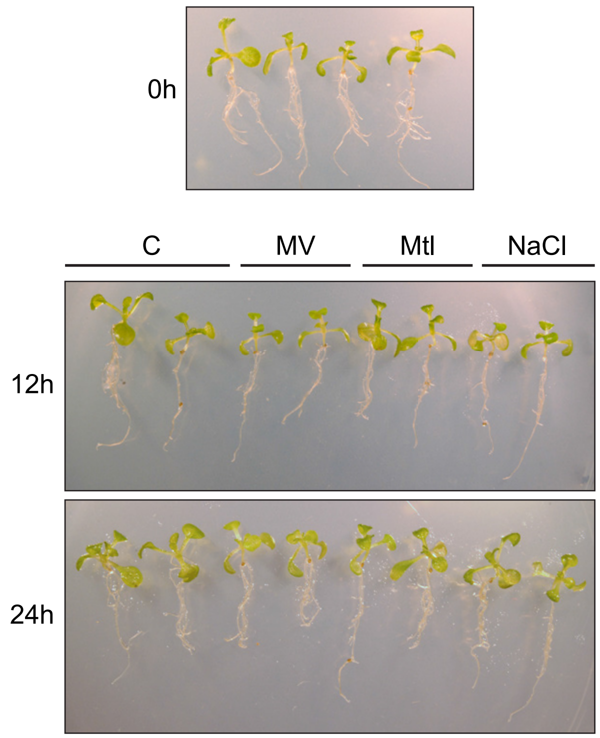

Figure S1. Oxidative, osmotic, and salt stress treatment of PAG1-FLAG seedlings. PAG1-FLAG seedlings were grown for 8 days after imbibition (DAI) on half-strength MS media supplemented with 1% sucrose and 0.4% agar (solid) at 22ºC and a 16-hour light (yellow) / 8-hour dark (blue) cycle. Seedlings were transferred to MS + 1% sucrose + 0.1% agar (liquid) for a 48-hour acclimation period, after which the appropriate stressor was added: 10 μM methyl viologen (MV) to induce oxidative stress, 300 mM mannitol (Mtl) for osmotic stress, and 150 mM NaCl for salt stress. Samples treated for 12- and 24 hours (12h and 24h) appeared very similar to the untreated samples (C) or the ones at the begining of treatment (0h) and were taken for sample preparation.

Figure S2

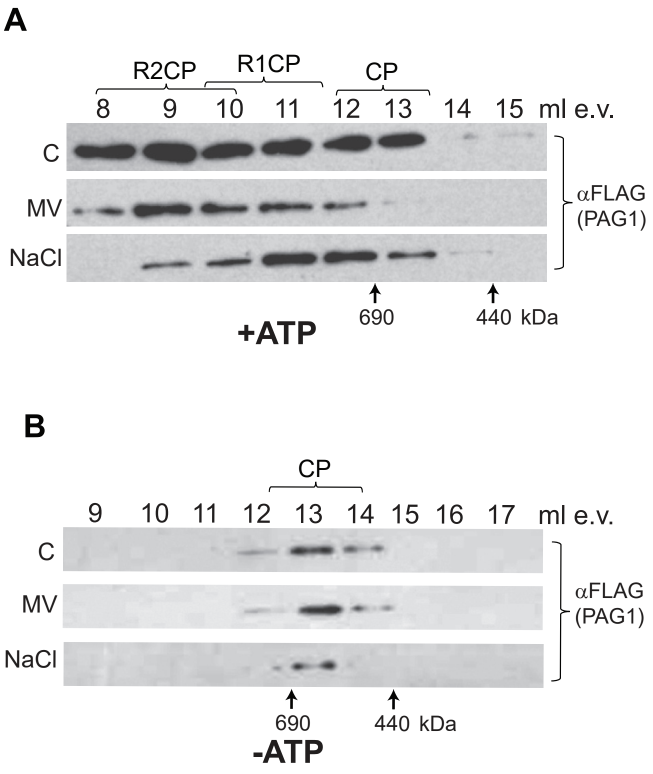

Figure S2. Size exclusion chromatography (SEC) analysis of total cell lysates from seedlings grown on non-stress (C), methyl viologen (MV) or salt (NaCl) stressed media. The cell lysates were made in the presence of 20mM ATP to preserve the association of RP-CP into 26S proteasome (**A**), or in the absence of ATP to disfavour the 26S holoenzyme formation (**B**). Lysates were run on GE Healthcare AKTA purifier 25 with Superose™ 6 10/300 GL column. Eluants were collected in 0.5 ml fractions and resolved on SDS-PAGE followed by immunoblotting with anti-FLAG antibody for PAG1-containing complexes that primarily represent either 20S CP or 26S holoenzymes. The approximate molecular weights corresponding to a few different elution volumes (e.v.) for reference were labelled under the blots.

Figure S3.

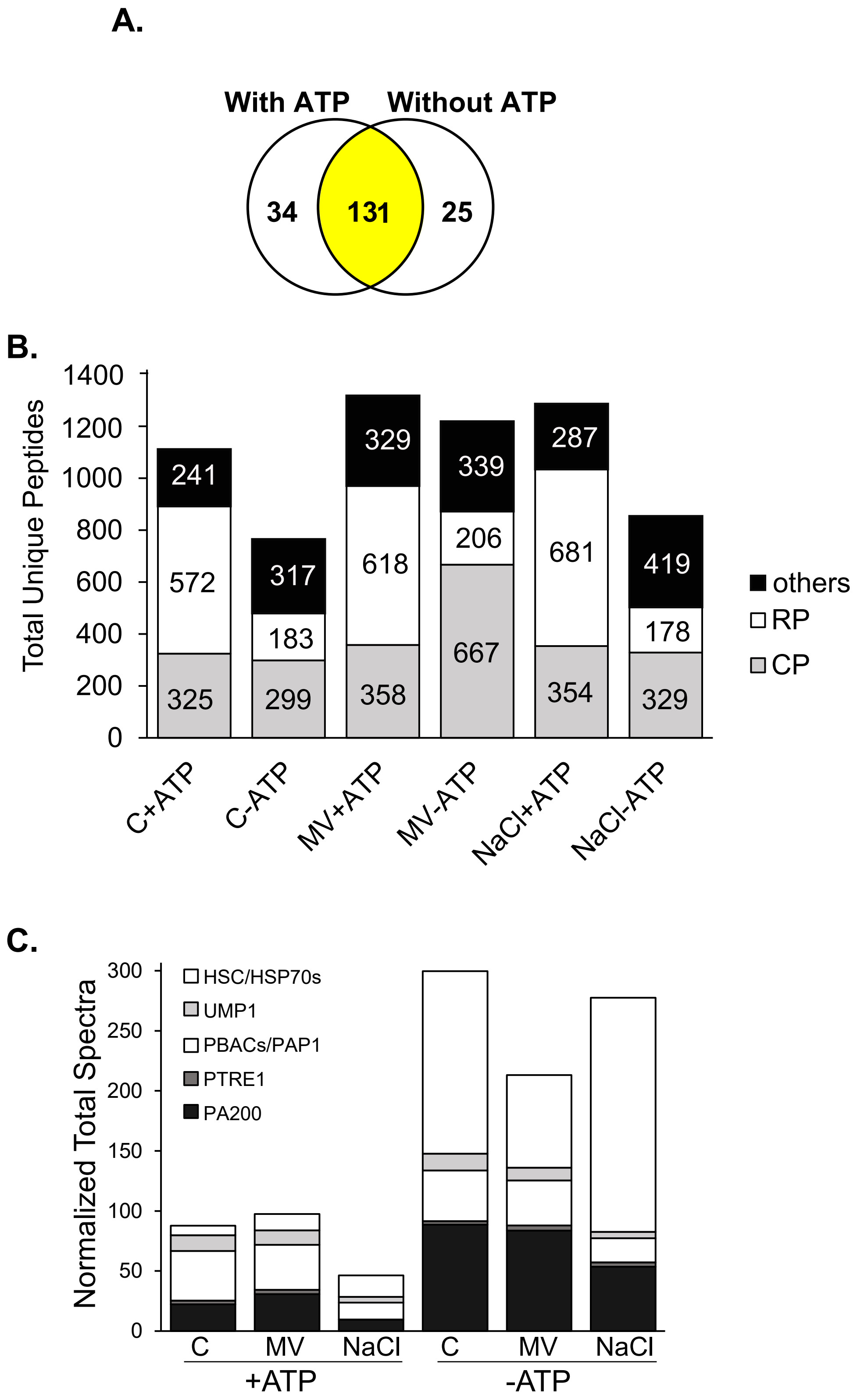

Figure S3. Summary of identified proteins and unique peptides by LC-MS/MS analysis.
A. Total number of proteins identified with at least two exclusive unique peptides from at least one out of the six total purified proteasome samples. 131 common proteins were identified in the two categories with and without ATP regardless of stress treatments.
B. Total number of unique peptides identified from all CP and RP subcomplex subunits and from the other proteins associated with the purified proteasome complexes.
C. Normalized total peptide spectra identified by LC-MS/MS for PAPs. This is a graphic representation of the Supplementary Table S4.

Figure S4.

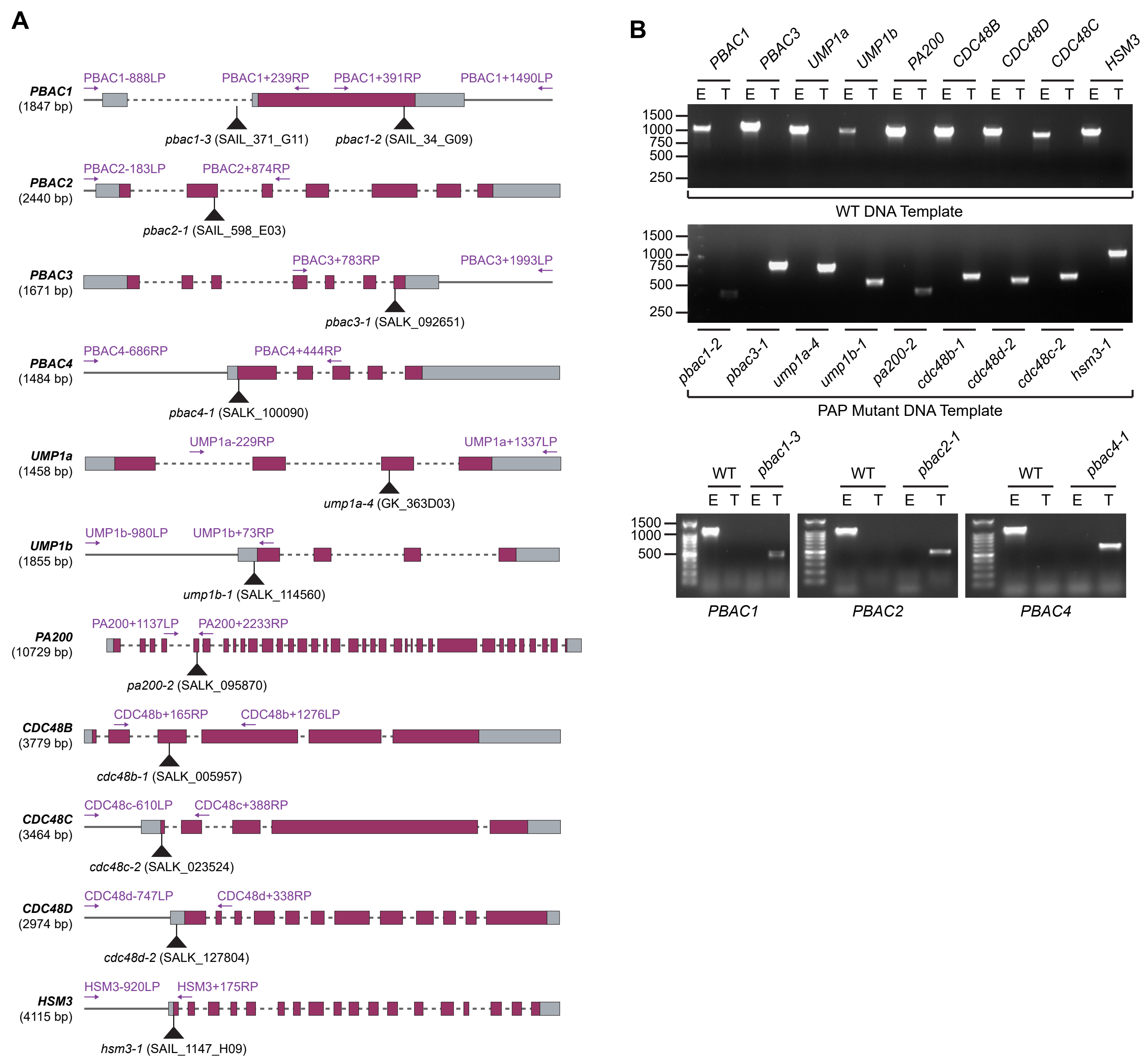

Figure S4. Confirmation of T-DNA insert homozygosity within proteasome-associated protein (PAP) mutants.

A. Diagrams of Arabidopsis *PBAC1-4*, *UMP1a/b*, *PA200*, *CDC48B-D* and *HSM3* genes, encoding selected PAPs. Magenta boxes represent coding regions, grey boxes represent untranslated regions, dotted lines represent introns, and solid lines represent intergenic regions. SAIL, SALK, and GABI-Kat (GK) lines with T-DNA insertions in the 5'UTR or exon of PAP genes were obtained from the Arabidopsis Biological Resource Centre (ABRC). Predicted T-DNA insertion sites are marked by black triangles while binding sites for primers used in PCR genotyping are marked by purple arrows.

B. Corresponding PCR products resolved on a 1% agarose gel. As shown in (**A**), primer pairs were designed to amplify a segment of the endogenous gene (E) bordering the insert, as well as a segment of the T-DNA (T). Genomic DNA extracted from flower buds of each PAP mutant was used to demonstrate homozygosity by the absence of an E product and the presence of a T product. Wild-type (WT) (Col-0) genomic DNA was used as a positive control.

Figure S5.

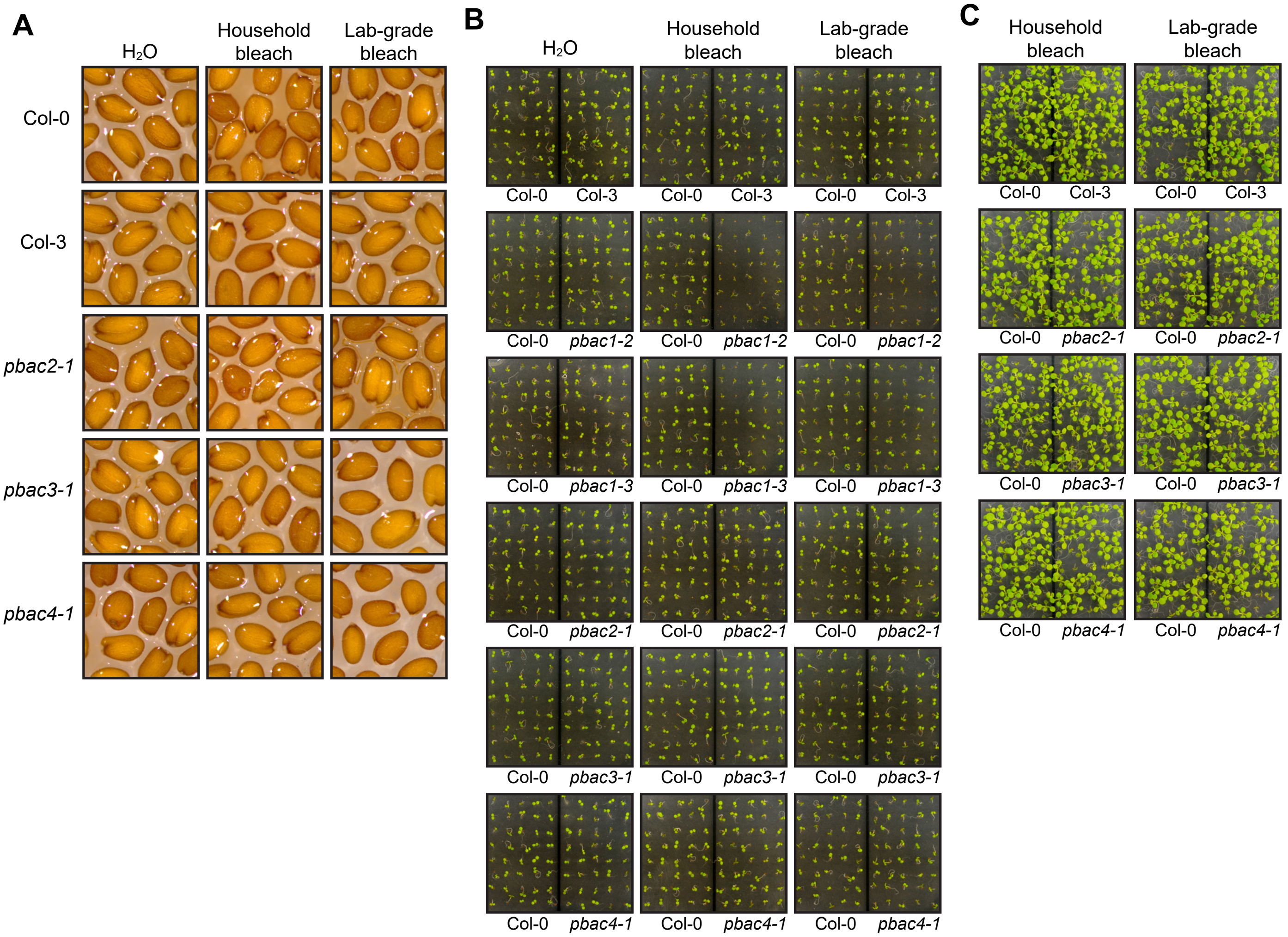

Figure S5. Appearance of all *pbac* mutant seeds, and their subsequent development after treatment with bleach. Dry Col-0, Col-3, *pbac2-1*, *pbac3-1*, and *pbac4-1* seeds were treated with either ddH2O (non-sterile), household bleach (LAVO-12; 2.58% NaOCl), or laboratory-grade bleach (BioShop; 2.58% NaOCl). (**A**) Images of seeds immediately following treatment, for lines which did not show a prominent seed coat darkening or tearing phenotype. (**B**) Images of all treated pbac mutant seeds sown on half-strength MS, 4 days after imbibition (DAI). (**C**) Images of the household and lab-grade bleach-treated seeds shown in (**A**), after growth on half-strength MS for 10 DAI. *pbac1-2* and *pbac1-3* mutations are in the Col-3 genetic background. The remainder of the mutants are of the Col-0 ecotype. Note that non-sterile (H_2O) seeds could not be grown past 5 DAI due to the appearance of contamination.

Figure S6.

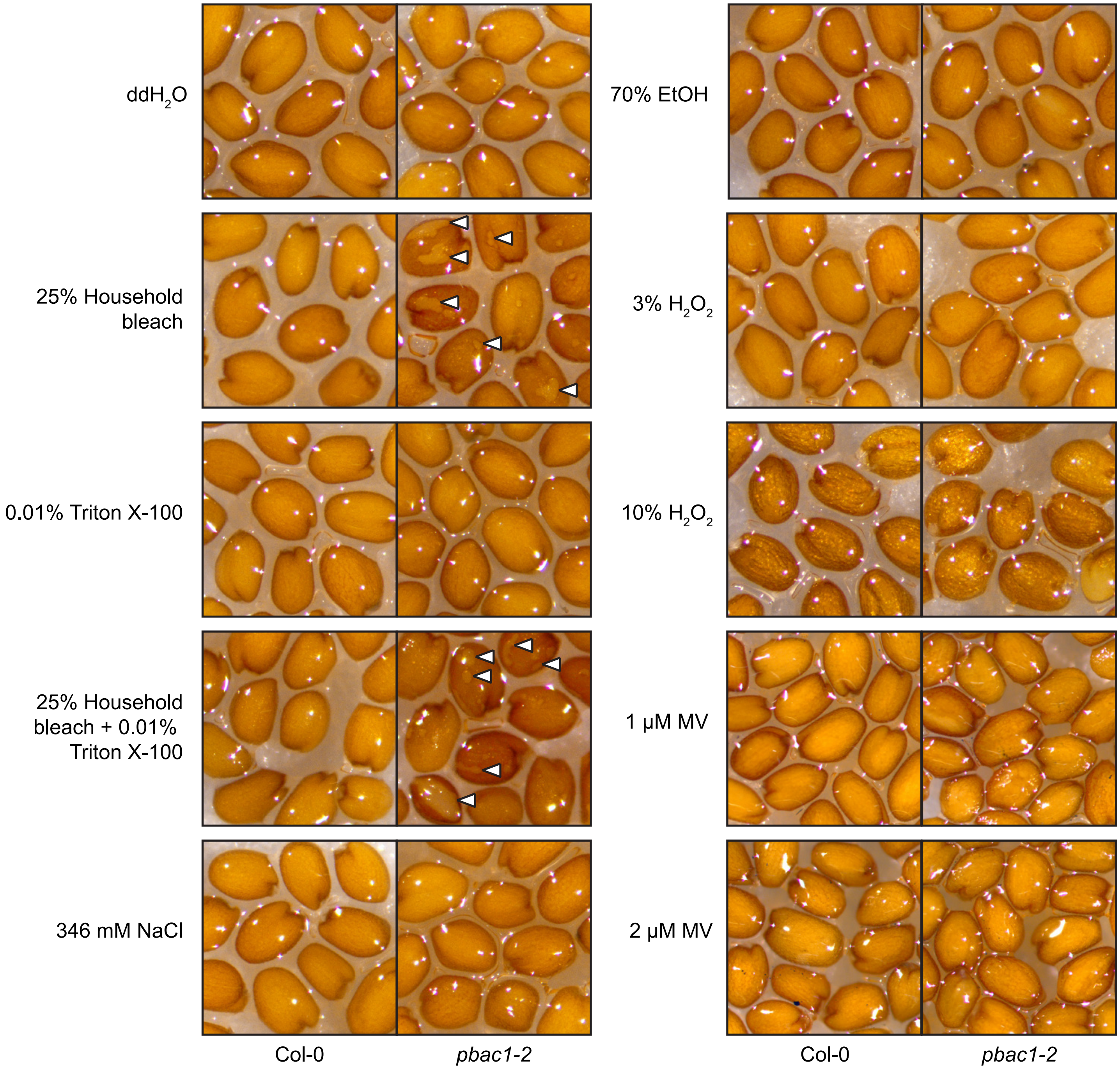

Figure S6. Effect of various chemical pre-treatments on the appearance of *pbac1-2* seeds.
Dry seeds were treated with a variety of sterilization and oxidative stress agents for 10 min, including household bleach (LAVO-12; 2.58% NaOCl final concentration), Triton X-100, ethanol (EtOH), hydrogen peroxide (H_2O_2), and methyl viologen (MV). 346 mM NaCl served as a control for the effect of sodium on seeds as it contains the same concentration of Na+ ions as 2.58% NaOCl. Magnified images of WT (Col-0) and *pbac1-2* seeds immediately after each chemical treatment are shown, highlighting changes to the appearance of the testae. White arrowheads point towards holes in the seed coats.

Figure S7

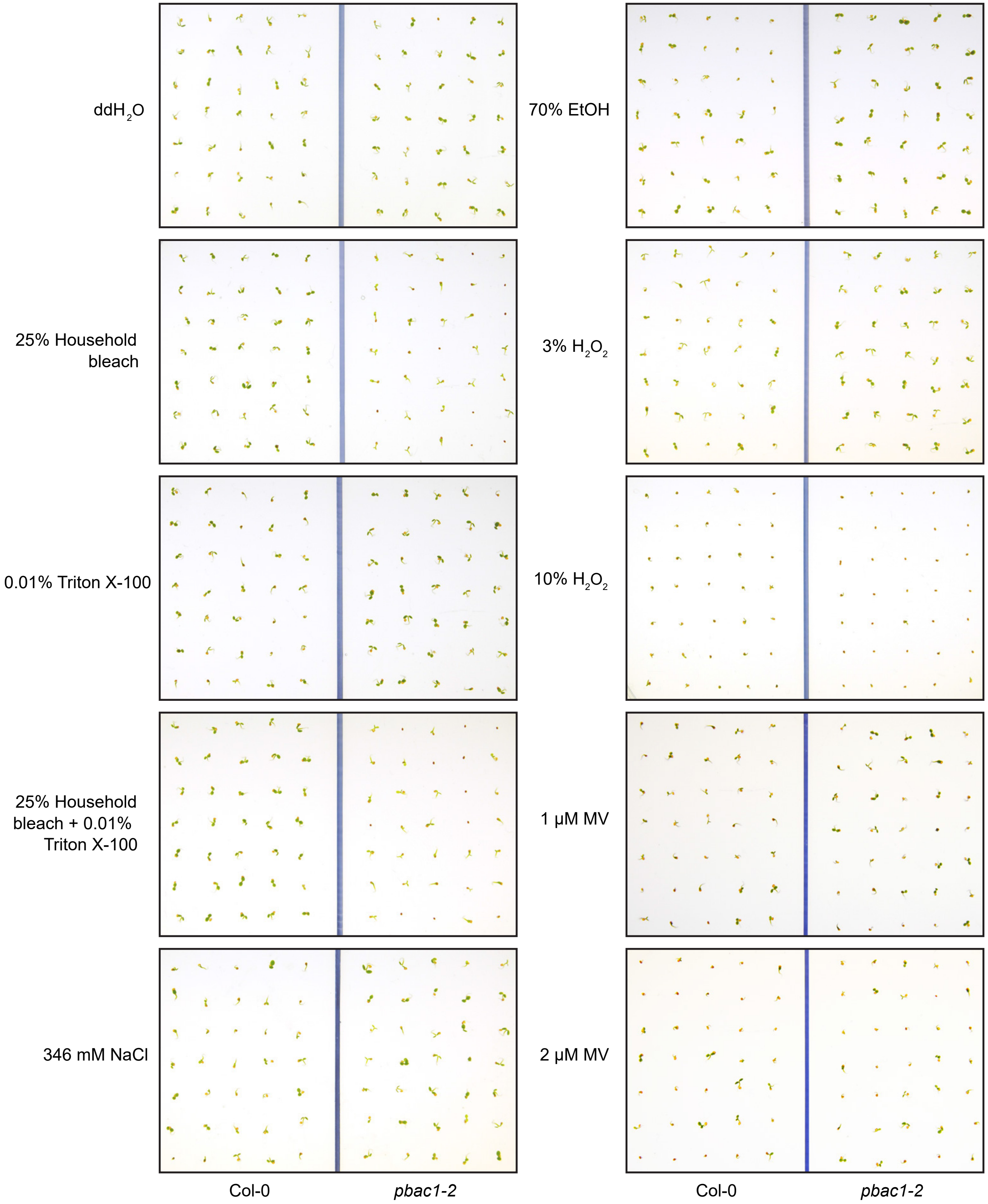

Figure S7. Effect of various chemical pre-treatments on the appearance and subsequent germination of *pbac1-2* seeds. Dry seeds were treated with a variety of sterilization and oxidative stress agents for 10 min, including household bleach (LAVO-12; 2.58% NaOCl final concentration), Triton X-100, ethanol (EtOH), hydrogen peroxide (H_2O_2), and methyl viologen (MV). 346 mM NaCl served as a control for the effect of sodium on seeds as it contains the same concentration of Na+ ions as 2.58% NaOCl. Images of treated WT and *pbac1-2* seeds were taken two days after being sown on half-strength MS media.

Figure S8.

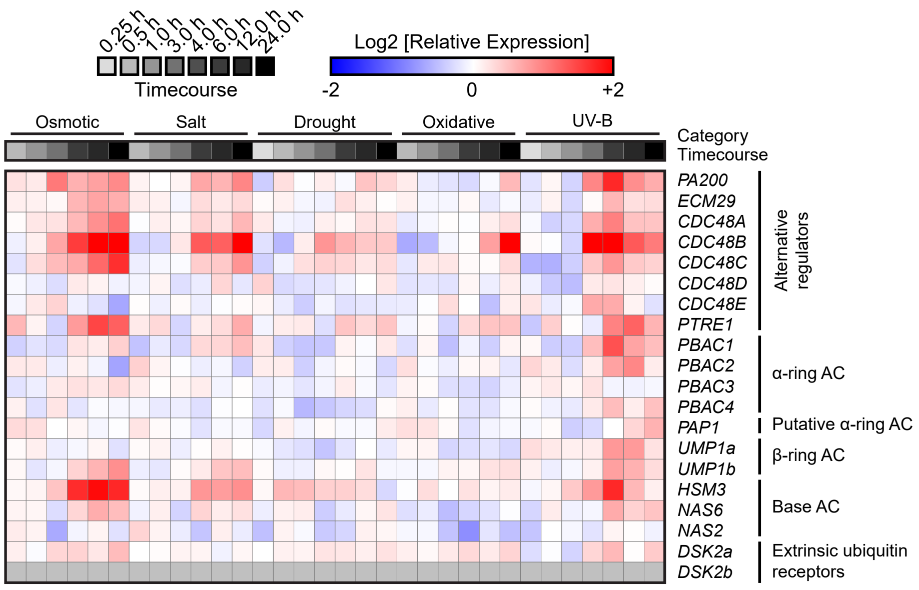

Figure S8. Expression of Arabidopsis PAP genes under abiotic stresses.
Microarray data of Arabidopsis seedlings exposed to cold (4°C), osmotic (300 mM mannitol), salt (150 mM NaCl), drought (air stream), genotoxic (1.5 µg/ml bleomycin), oxidative (10 µM methyl viologen), UV-B, wounding, and heat shock (38°C) stresses were obtained via the BAR Expression Browser (http://bar.utoronto.ca/) and organized into a heat map using Morpheus (broadinstitute.org). Heat map displays log2-transformed expression-fold changes with min/max cut-off values of -2/+2. Note that for heat stress, the 4.0- to 24.0-hour time points represent variable 25°C recovery times (Rcvry) following an initial 3.0-hour heat shock (HS) at 38oC. Relative expression is illustrated with a cut-off value of 2.0. Grey squares indicate that data is not available for a particular gene.
