# Supplementary material for: Oxidative and salt stresses alter the 26S proteasome holoenzyme and associated protein profiles in Arabidopsis thaliana
Source: BMC Plant Biol. 2021 Oct 25;21:486. doi: 10.1186/s12870-021-03234-9 (PMC8543921; doi:10.1186/s12870-021-03234-9)
Supplement: Supplementary file 6 — Additional file 6. [file 12870_2021_3234_MOESM6_ESM.pdf]

Original blots used for Figure 1B

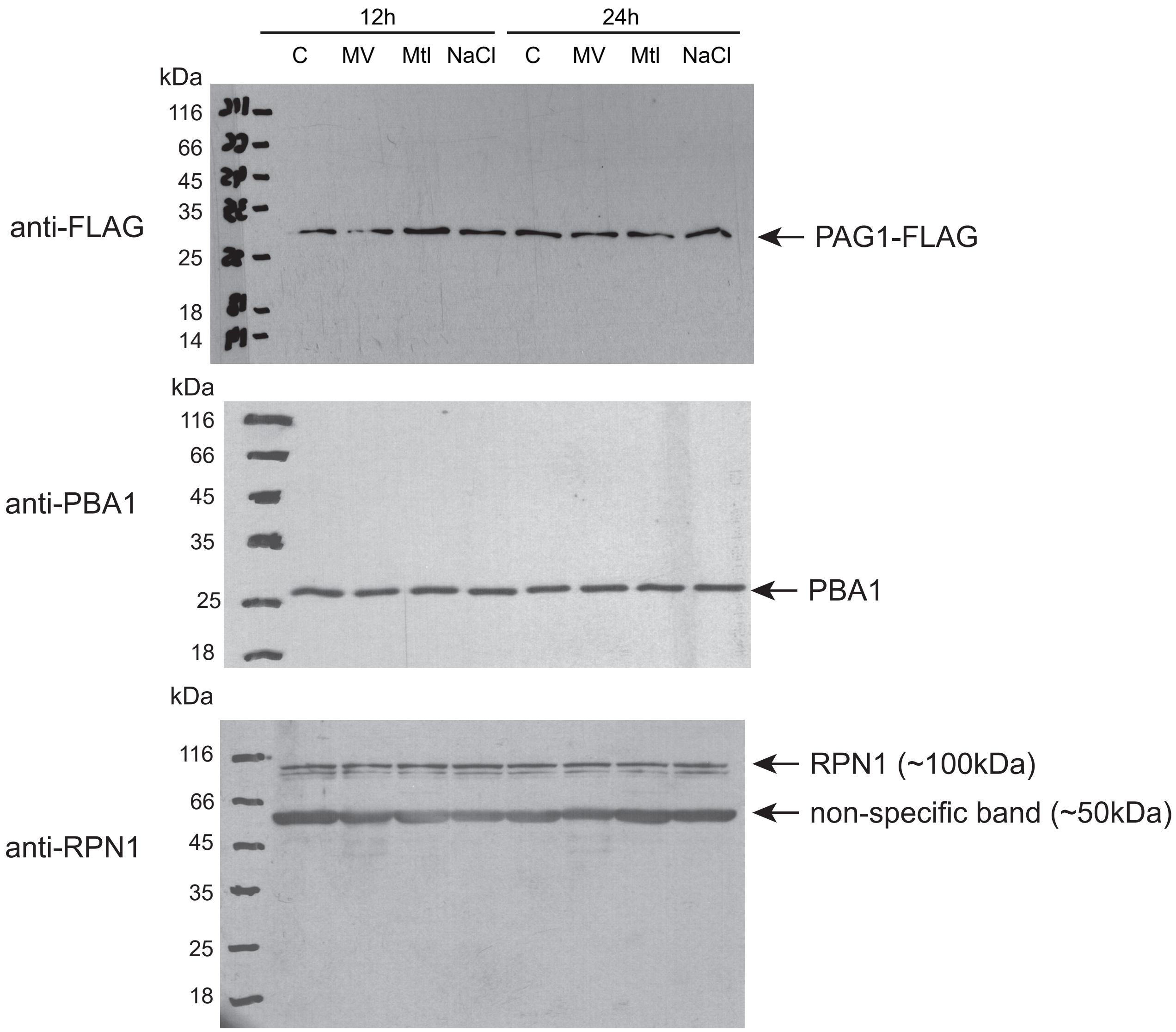

Original immunoblotting images used in Figure 1B. Note the strong bands in anti-RPN1 blot are non-specific bands and the top ~100kDa bands represent the correct RPN1 proteins. The weaker bands right under the RPN1 may represent partially degraded products.

Original blots for Figure 2

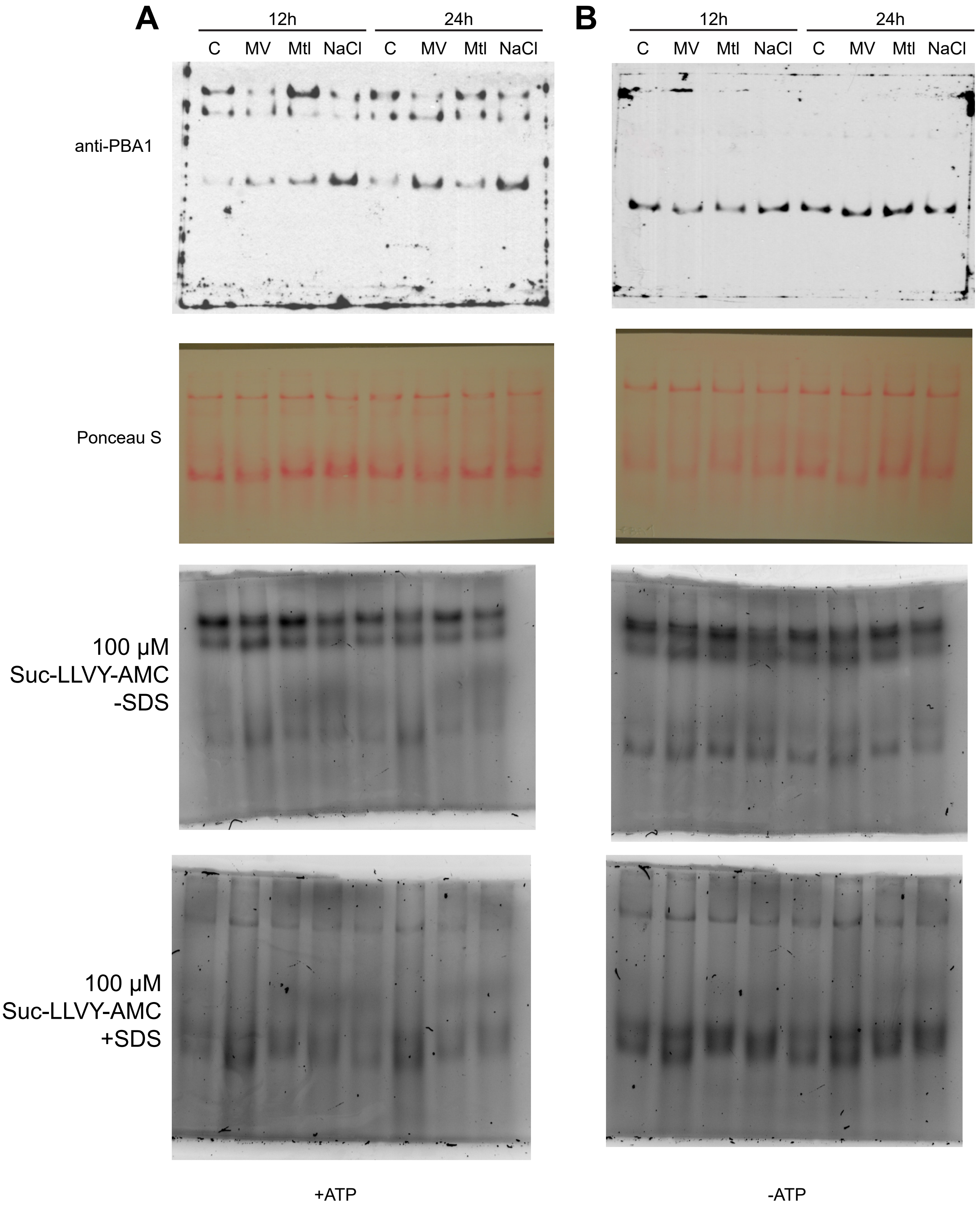

Original in-gel peptidase assay results and Ponceau S staining images of the native-PAGE before blotting with anti-PBA1 antibody in Figure 2.

Original immuoblotting images in Figure 3E.

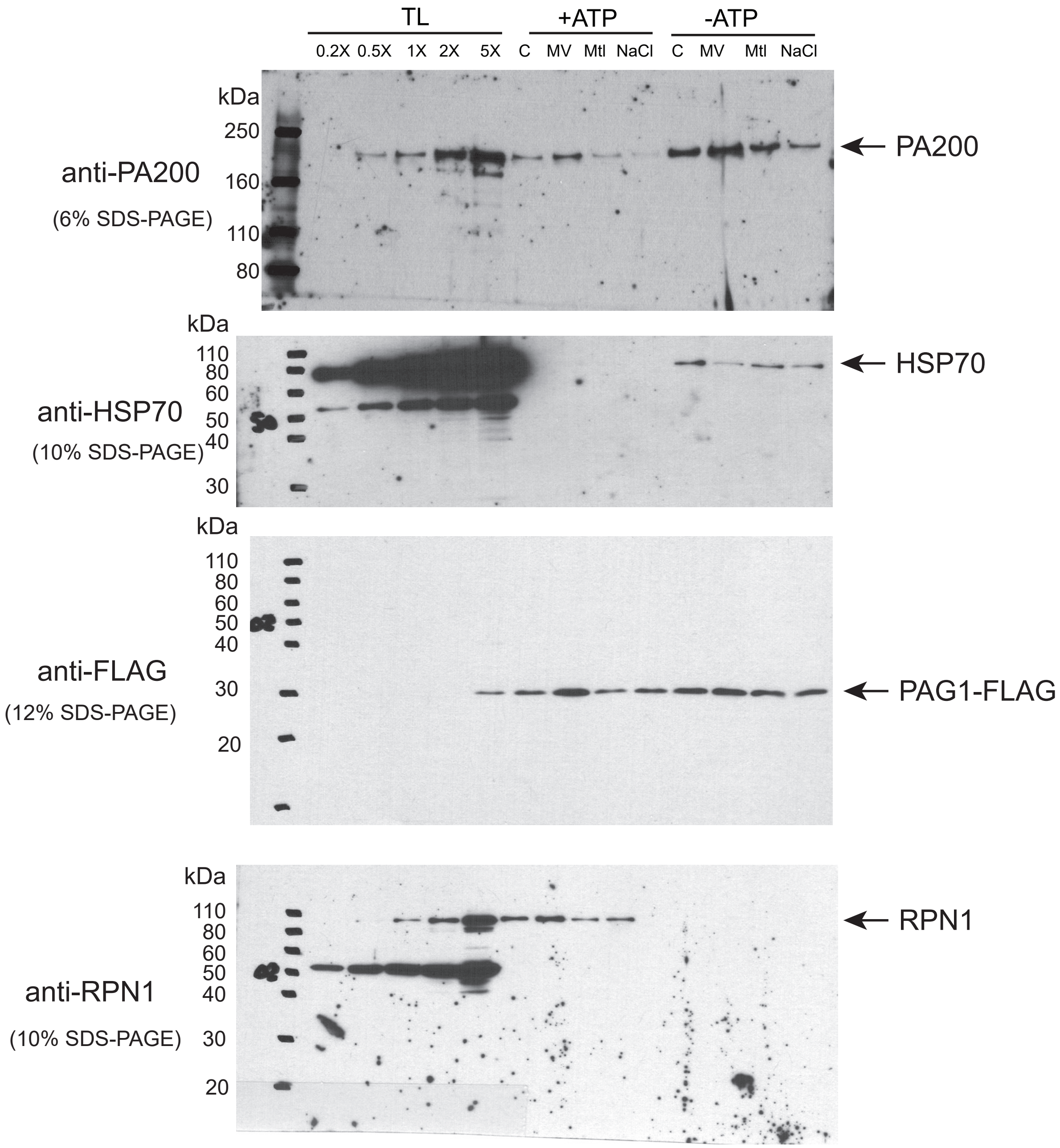

Original immuoblotting images in Figure 3E. Please note the samples are only labelled once on the very top and all four blots have the sample loading order.

Original blotting images in Figure 5A

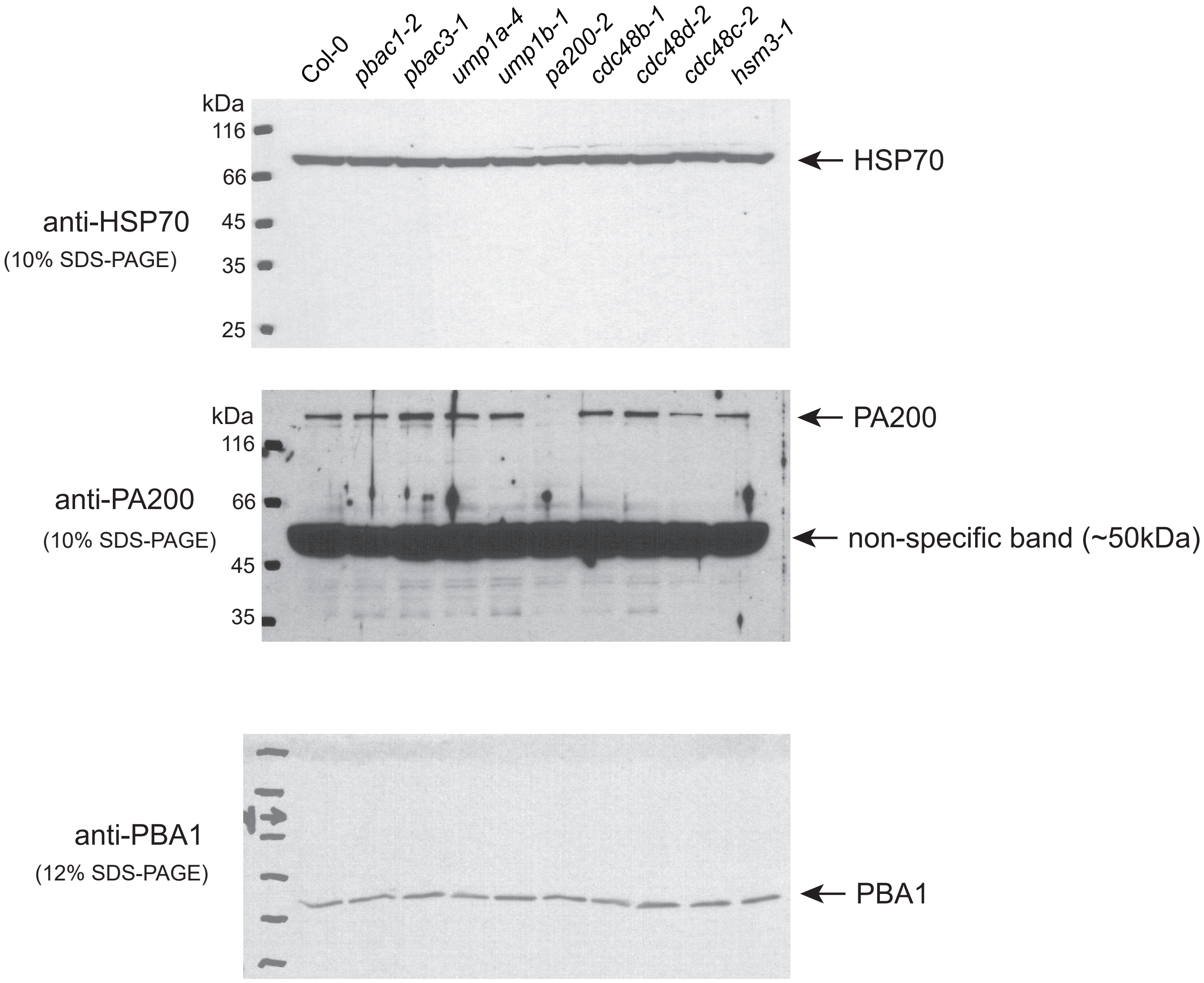

Original blotting images in Figure 5A. Please note the samples were only labelled once on the very top and all three blots have the sample loading order.

Original blotting images used in Supplemental Figure S2.

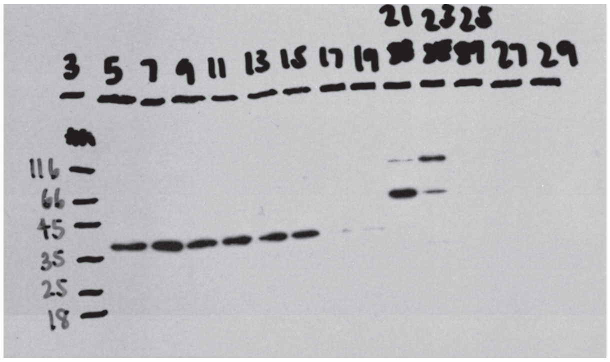

MS+ATP

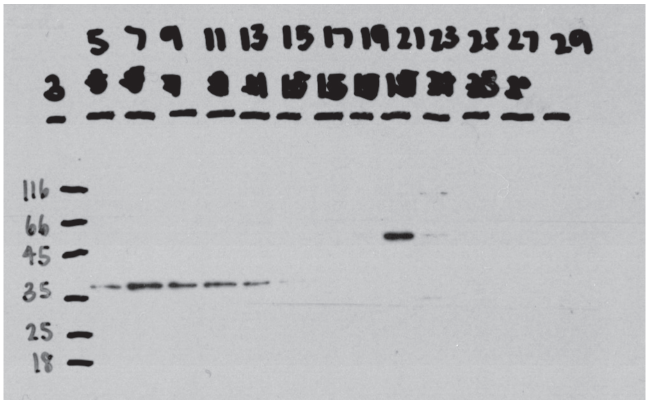

MV+ATP

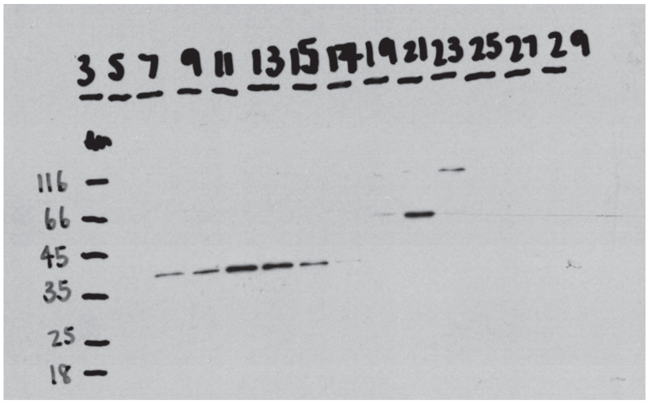

NaCl +ATP

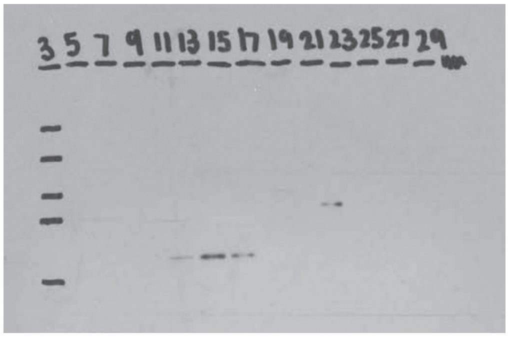

MS-ATP

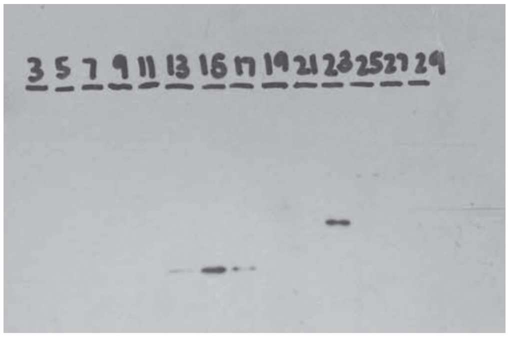

MV-ATP

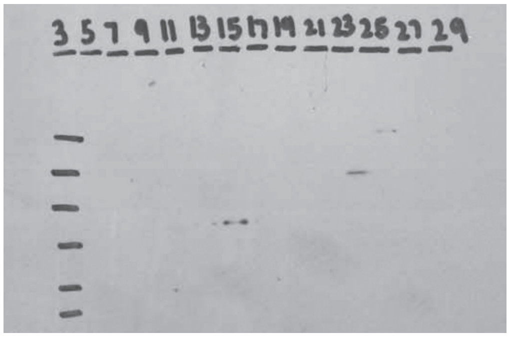

NaCl -ATP

Original Anti-FLAG antibody immunoblot on size exclusion fractions used in supplemental Figure S2. The fractions were converted to corresponding elution volumes and indicated in the final Figure S2.

Original agarose gel images used in Figure S4B

(top and middle) Left to right: pbac1-2, pbac3-1, ump1a-4, ump1b-1, pa200-2, cdc48b-1, cdc48d-2, cdc48c-2, hsm3-1. In pairs of endogenous gene (left) and T-DNA insert (right)

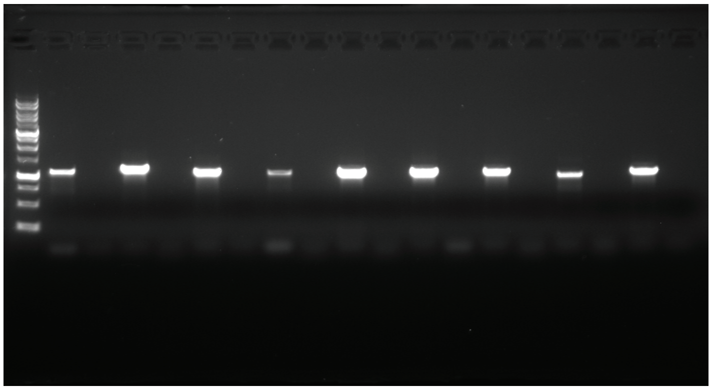

Top

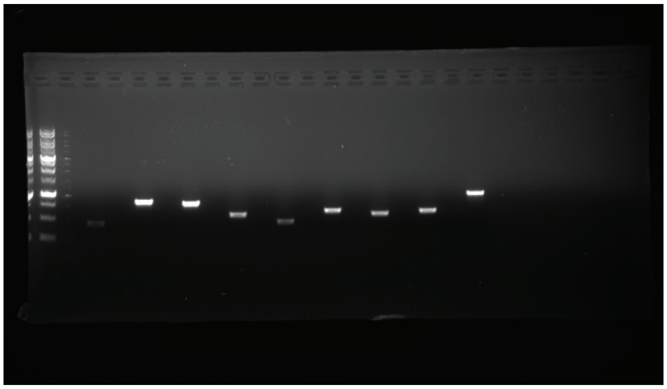

Middle

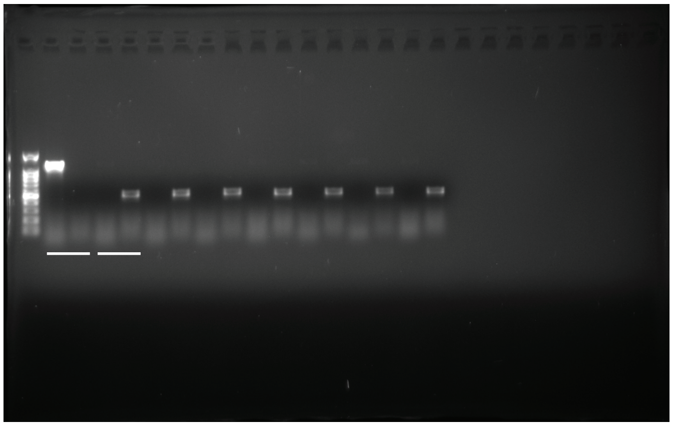

Bottom left: WT and *pbac1-3* (x7)
The WT and the identified correct mutant used in this study is underlined.

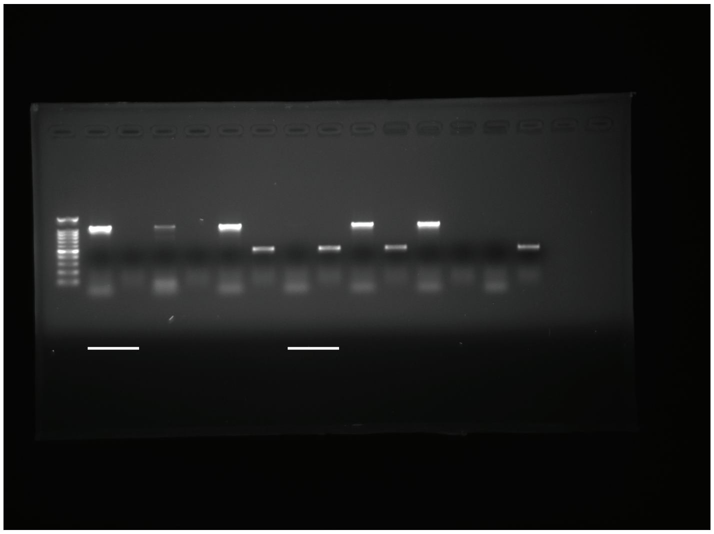

Bottom middle: WT and *pbac2-1* (x6)
The WT and the identified correct mutant used in this study is underlined.

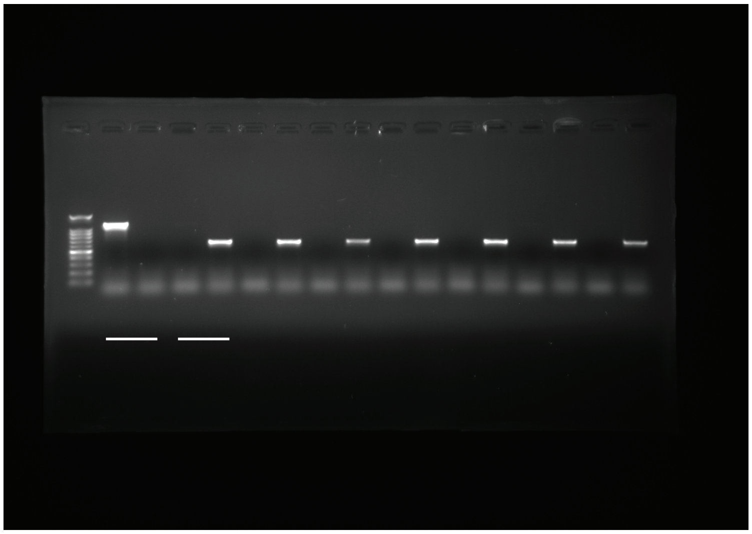

Bottom right: WT and *pbac4-1* (x7)
The WT and the identified correct mutant used in this study is underlined.
